# Environmental, climatic, and social risk factors of severe fever with thrombocytopenia syndrome and the implications of climate change

**DOI:** 10.1016/j.onehlt.2026.101430

**Published:** 2026-04-26

**Authors:** Yating Yang, Jiangping Ren, Guoyou Qin, Yihan Lu, Ying Liu, Jimin Sun, Ye Yao, Yang Liu

**Affiliations:** a Department of Biostatistics, National Commission of Health Key Laboratory for Health Technology Assessment, and the Key Laboratory of Public Health Safety of Ministry of Education, School of Public Health, Fudan University, Shanghai, China; b Zhejiang Key Lab of Vaccine, Infectious Disease Prevention and Control, Zhejiang Provincial Center for Disease Control and Prevention, Hangzhou, China; c Shanghai Institute of Infectious Disease and Biosecurity, Fudan University, Shanghai, China; d Department of Epidemiology, Ministry of Education Key Laboratory of Public Health Safety, School of Public Health, Fudan University, Shanghai, China; e PATH, London, United Kingdom; f Centre for Mathematical Modelling of Infectious Diseases, London School of Hygiene & Tropical Medicine, London, United Kingdom; g Centre on Climate Change and Planetary Health, London School of Hygiene & Tropical Medicine, London, United Kingdom; h Department of Infectious Disease Epidemiology and Dynamics, Faculty of Epidemiology and Population Health, London School of Hygiene & Tropical Medicine, London, United Kingdom

**Keywords:** Severe fever with thrombocytopenia syndrome, Climate change, Human mobility, Socio-environmental factors, Projections

## Abstract

**Background:**

SFTS is a tick-borne infectious disease with a high case fatality ratio with geographic expansion. While there have been some studies exploring the link between environmental and climatic factors on SFTS, we further test a hypothesis that social factors may play a role in the increasing burden.

**Methods:**

Using data from Zhejiang Province, China (2011−2023), we estimated the effects of environmental, climatic, and social factors on SFTS incidence using a Bayesian negative binomial regression model. Rigorous model selection processes, including variations of functional formats among independent variables and model validation, were followed. Influences of each covariate were assessed using block cross-validation experiments. We predicted the implications of the associations derived until 2028 based on climate projections.

**Findings:**

Environmental, climatic, and social factors were all selected in the final model. Higher risk areas included mountainous and cropland regions, with moderate temperatures, high precipitation, and longer sunlight exposure. Urbanization is negatively associated with SFTS risks, while human mobility is positively associated with SFTS risks. Spatial random effects do not significantly improve model performance after including human mobility measures and were thus excluded from the final model. By 2028, we are expecting a 23% increase in SFTS incidence; 9% more counties are likely going to report their first case. An SFTS season is expected to expand from March to October to year-round.

**Conclusions:**

This study provides a comprehensive investigation of the associations between environmental, climatic, and social factors and SFTS risks using a data-driven approach. Our findings underscore the growing role of social dynamics, particularly mobility, in shaping SFTS risk. SFTS in Zhejiang is expected to spread wider and last longer, calling for timely adaptation of public health strategies.

## Highlights

• The interplay of geography, climate, and society drives SFTS risk expansion.
• Human mobility increases the spread of tick-borne SFTS across urban and rural areas.
• Climate change is predicted to expand SFTS risk to new regions and seasons by 2028.
• SFTS is emerging in cities, challenging its conventional view as a rural disease.

## 1 Introduction

Severe Fever with Thrombocytopenia Syndrome (SFTS) is an emerging tick-borne disease caused by the SFTS virus, primarily transmitted by *Haemaphysalis longicornis* [1]. It may also spread via contact with infected farm or domesticated animals or, less commonly, through human-to-human contact [2]. With a high case fatality ratio (2.7% to 45.7%, mean 10.5%) and no effective pharmaceutical interventions [2], SFTS remains a serious emerging public health threat [3]. Since its first identification in China in 2009, human cases have been reported in South Korea [4], Japan [5], Southeast Asia [6], [7], [8], and the United States [9], [10], [11].

Although SFTS cases are concentrated in East Asia, the potential for introduction into other regions - especially where already burdened by vector-borne diseases, such as Southeast Asia and North America [1], [12], [13]– highlights the global relevance to understanding SFTS epidemiology.

China bears the world's largest SFTS burden, with an increasing trend over the past decade [14]. Environmental and climatic factors (e.g. temperature, elevation, and land use) have been considered the main drivers of SFTS given their close links to tick ecology [14], [15], [16]. The role of social factors, such as urbanicity and human mobility, has been under-explored [17], [18]. In this study, we aim at identifying the links between environmental, climatic, and social risk factors and SFTS transmission using a data-driven approach using data from Zhejiang province (2011–2023), China. Zhejiang Province represents one of the seven core endemic regions for SFTS in China, where nearly 99% confirmed cases nationwide have been concentrated since 2010 [19]. The province has consistently ranked among the top six in national incidence, with reported cases showing a substantial increasing trend over the past decade [20]. Notably, Zhejiang also reports the highest case-fatality rate nationwide (average CFR ≈ 11.1% from 2010 to 2023), underscoring its substantial contribution to the national SFTS burden [20], [21]. Moreover, recent reports of human-to-human transmission clusters, including healthcare-associated infections in 2023, further highlight the urgency and regional importance of conducting an in-depth investigation in this province [22], [23].

While SFTS is conventionally considered a rural health issue [24], recent detections of SFTSV in urban stray animals in China [25], [26], [27], South Korea [28], and Thailand [29] offer alarming evidence that leads us to reconsider. Furthermore, evidence on the role of human mobility in tick-borne disease transmission has been inconsistent [30]. Some studies argue that ticks' limited life-time movement range makes spatial spillover negligible [30]. Spatial autocorrelation of SFTS incidence is therefore considered weak. Others argue that human mobility patterns may have changed the ways humans, vectors, and animal hosts encounter each other [2], [31], making the introduction to new regions a highly likely scenario [32]. In this study, we test the hypothesis that spatial autocorrelation and human mobility may help better predict SFTS transmission.

In the context of global climate change, a comprehensive understanding of how the environment, climate, and social risk factors affect SFTS burden is urgently needed [18], [33]. With the associations identified in this study, we project the future trends of SFTS with spatial and temporal nuances. The evidence generated in this study would inform future public health surveillance and intervention designs in the face of emerging SFTS risks.

## 2 Methods

### 2.1 Study population

SFTS is a notifiable disease in mainland China. Healthcare providers and public health agencies are required to report any case (either clinically or laboratory confirmed, see supplementary method 1.1 for more details) to the China Information System for Disease Control and Prevention (CISDCP) within 24 h after diagnosis. This study relies on county-level (i.e. administrative level three, ADM03) SFTS cases reported in Zhejiang via this system between January 1, 2011, and December 31, 2023. Zhejiang is a coastal province in the southeast of China with a humid and subtropical climate and has a population of approximately 66 million [34].

### 2.2 Potential factors affecting SFTS transmission dynamics

A comprehensive literature review was conducted to identify potential geographical, climatic, and socio-environmental covariates associated with SFTS. The ecological rationale and supporting references for each variable are summarized in Table S1. Meteorological variables included mean temperature, cumulative precipitation, relative humidity, and sunshine duration, as these factors have been consistently reported to influence tick survival, seasonal activity, and SFTS transmission dynamics (Table S1). Wind speed and atmospheric pressure were not included because existing evidence regarding their association with SFTS remains limited and inconsistent, and their biological mechanisms in tick-borne transmission are less clearly established compared with temperature- and moisture-related indicators [15], [35], [36], [37].

Based on existing literature, we included the following factors in the regression model: land cover [16], elevation [16], [38], and climate [36]. Land cover data was obtained from the China Land Cover Dataset (CLCD) [39]. We considered both the area (km^2^) and proportion(%) of cropland, forest, shrubland, and grassland. Elevation data was extracted from the Copernicus Digital Elevation Model (DEM) [40]. Historical climate data were obtained from the China Meteorological Data Service Center [41]. Future climate data were extracted from CMIP6 using the MRI-ESM2–0 general circulation model under the SSP2–4.5 scenario [42]. We applied mean bias correction to temperature data based on prior validation results [43] (Supplementary Method 1.2).

SFTS is a zoonotic disease [44] and therefore, any social risk factors that may influence the interface between humans, animal hosts, and vectors may be associated with SFTS transmission. In terms of social risk factors, we included urbanicity and human mobility. Urbanicity was characterized by the proportions of populations living in urban settings and was calculated using data from the Zhejiang Statistical Yearbook [34]. Mobility was described by two metrics: highway mileage [34] and mobility (as measured by Baidu Migration Index) (See supplementary method 1.3 for details). More specifically, mobility is a unitless measure that characterise the relative magnitude of inter-regional travel.

All statistical analyses were conducted at a unified monthly scale; detailed data harmonization procedures for variables originally collected at different temporal resolutions are provided in supplementary method 2. To assess the robustness of the within-year stability assumption for annual variables, we performed a sensitivity analysis using natural cubic spline interpolation to generate smooth monthly estimates from annual values. The spline-based specification allowed gradual intra-annual variation in geographical and socio-environmental indicator (see supplementary method 4).

### 2.3 Statistical analyses

We applied a negative binomial regression model, fitted using Integrated Nested Laplace Approximation (INLA), to assess the link between SFTS disease burden and environmental, climatic, and social risk factors. The negative binomial specification was chosen because the outcome data exhibited overdispersion, with the variance exceeding the mean across county-month observations. To evaluate potential zero inflation and assess model robustness, we additionally fitted a zero-inflated negative binomial (ZINB) model as a sensitivity analysis. The results were comparable between the two specifications and did not materially alter the substantive conclusions (see supplementary method 4).

In the development process, we first tested the association between potential factors and SFTS disease burden using a univariate model while varying assumptions around their function form (e.g. original scale, log-transformed, spline functions) and summarising methods (i.e. lagged or cumulative values over six months). The goodness of fit measures WAIC, DIC, and the logarithm of the pseudo marginal likelihood (LPML) [45] were used to evaluate these univariate models and only potential factors that improved model performance were included in the follow-up analysis(Supplementary Text 2). Then, multicollinearity among independent variables was assessed. Those with a variance inflation factor (VIF) greater than four were excluded from further analysis [46]. We further tested whether including spatial and temporal random effects could improve the model's performance using five-fold cross-validation. The spatial random effects were modelled using an intrinsic conditional autoregression model [47]. The temporal random effects were modelled using an ordinal categorical variable, *year*. More details on the model development process may be found in Supplemental Methods 2 and Fig. S1. Regional stratified analysis (i.e. dividing Zhejiang into southern, central, and northern parts) was used to demonstrate the robustness of our results.

To disentangle the relative importance of potential risk, we implemented a leave-one-out modelling exercise and calculated their corresponding out-of-sample (OOS) prediction errors using a five-fold spatiotemporal block-holdout cross-validation approach (see supplemental method 3.1) [48]. Changes in root mean square errors (RMSE) were used to characterise the relative contribution. Large and positive changes in RMSE when excluding a certain factor imply a large relative contribution. This process was repeated ten times for each factor to account for uncertainty. Additionally, we calculated the variance explained by risk factor groups, which quantifies the variance explained (see supplemental method 3.2).

### 2.4 Future projections

We generated future projections by 2028 to understand the implications of the associations we find in the context of climate change. Linear extrapolations of climate factors were used as a sensitivity analysis. For environmental and social risk factors, we applied linear extrapolation based on historical trends to reflect projected changes in population distribution and socioeconomic conditions, and incorporated these into the prediction model. To avoid overfitting, we refit the regression model using truncated time series: 2011–2020, 2011–2021, 2011–2022, and 2011–2023.

### 2.5 Defining an outbreak and season

SFTS is an emerging climate-sensitive infectious disease with no consensus defining an outbreak. Given its relatively low incidence and heterogeneous spatial distribution, outbreak definitions are often context-specific [14], [49]. In this study, we tested a range of outbreak thresholds while discussing the current and future trends of SFTS seasonality: 2, 5, and 10 per 100 million population-month to represent different transmission intensity levels. An SFTS season is defined as the period between the first and last time these outbreak thresholds were crossed in calendar year [14]. To further assess robustness, we additionally applied an alternative distribution-based definition of epidemic season. Specifically, the epidemic season was defined as the shortest continuous interval during which the cumulative case numbers accounted for more than 70%, 80%, or 90% of the total annual cases (see supplemental method 4). This complementary definition captures the main transmission window independent of predefined incidence cutoffs. Using both incidence-based and cumulative distribution–based definitions allowed us to comprehensively evaluate seasonal patterns and ensure the robustness of projected seasonal changes.

All analyses were performed in R version 4.4.1. Code is available at: https://github.com/YatingYang8/SFTS.git.

## 3 Results

**Fig. 1:**
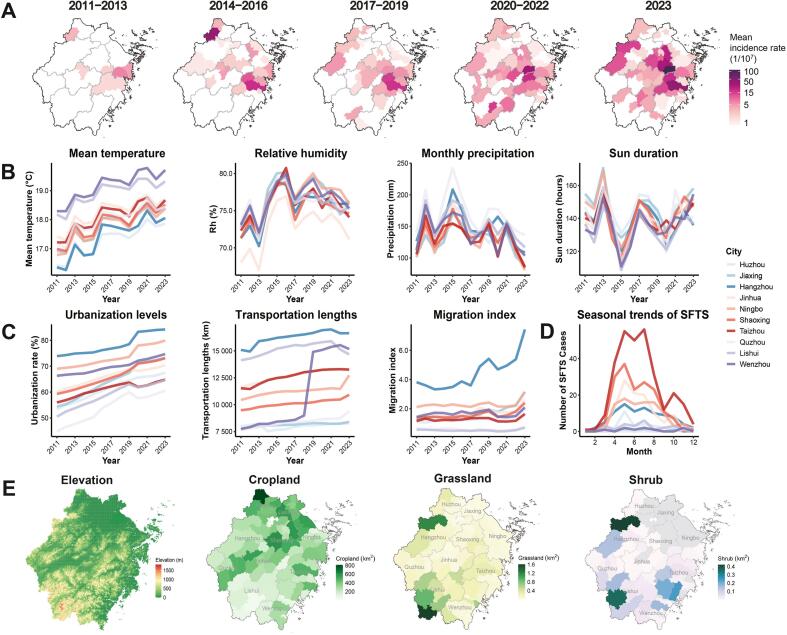
Temporal trends and geographic distribution of SFTS incidence in Zhejiang, China. All primary analyses were performed at the county level and that city-level reporting was used only for descriptive summarization and visualization. (A) The spatial distribution of mean SFTS incidence rate per 10,000,000 people in different counties over various time periods (log-transformed for visualization purposes). Darker colors represent higher incidence rates. (B) Annual trends of climatic factors (mean temperature [°C], relative humidity [%], monthly precipitation [mm], and sun duration [hours]) across different cities in Zhejiang from 2011 to 2023. (C) Annual trends of social factors (urbanization rates [%], transportation infrastructure [kilometers], migration index) in Zhejiang from 2011 to 2023. Missing data were imputed using the random forest multiple imputation algorithm. (D) Seasonal trends of SFTS for the entire study period across different cities in Zhejiang. (E) Spatial distribution of elevation, cropland, grassland, and shrub area in Zhejiang Province. The first panel shows an elevation map with 30 × 30 m^2^ resolution, where green (0–500 m) represents plains/low hills, yellow (500–1000 m) represents mountains, and red (>1500 m) represents plateaus. The remaining subplots show cropland, grassland, and shrub areas (km^2^), with darker colors indicating larger areas and gray representing missing data.

Between 2011 and 2023, Zhejiang province reported 823 SFTS cases, of which 112 eventually resulted in death. The overall case fatality ratio is 13.6% (95% confidence interval [CI]: 11.3–16.0%). Between 2011 and 2013, only ten counties reported SFTS cases; by 2023, 43 counties were reporting SFTS cases (Fig. 1A). The disease burden increased significantly over time (*p* < 0.05; Fig. S2), with most cases occurring between April and August (Fig. 1D).

We observed warming trends across Zhejiang while other climate factors remained relatively consistent (Fig. 1B). Urbanicity and human mobility increased between 2011 and 2023, with the sharpest increases observed in Hangzhou, the capital city of Zhejiang (Fig. 1C). The sharp increase in highway length per capita in 2018–19 in Wenzhou city was due to a development campaign to improve transportation infrastructure (Supplementary Text 1.3). Elevation is, on average, low in the northeastern cities as well as coastal regions, and on average, high in the southwestern cities. Overall, 70% of the province is covered by hills and mountains (>200 m) (Fig. 1E). Cropland, grassland, and shrub coverage showed minor variation over time (Fig. S3).

The literature basis and detailed screening process for covariate inclusion are summarized in Table S1 and Supplementary Text 2. Based on univariate model results, we applied a three-month lag on relative humidity, a one-month cumulative summary on sunshine duration, a two-month cumulative summary on monthly precipitation, and original time ranges for average temperature (Fig. S4). The best functional form of cropland coverage, urbanicity, highway lengths, and monthly precipitation is logarithmic (Fig. S5). Elevation, grassland coverage, shrub coverage, mean temperature, relative humidity, sun duration, and mobility were modelled using splines to capture nonlinear associations (Fig. S5). Forest coverage was highly correlated with elevation (*r* > 0.7). In preliminary univariate analyses, elevation demonstrated a stronger and more consistent association with SFTS incidence and yielded superior model fit statistics compared with forest coverage (Figs. S6–S7). Therefore, to reduce multicollinearity while preserving the more informative predictor, forest coverage was excluded from the multivariable model.

After adjusting for the risk factors above, an additional spatial random effect does not significantly improve the model fit (Supplementary Text 2.4, Table S2, Figs. S8–S10). This may imply that the spread of SFTS is either not spatially autocorrelated or that such autocorrelation has already been captured by other factors explicitly included in the regression model (i.e. mobility). Thus, the baseline model we present does not include a spatial random effect and only includes a temporal random effect term.

**Fig. 2:**
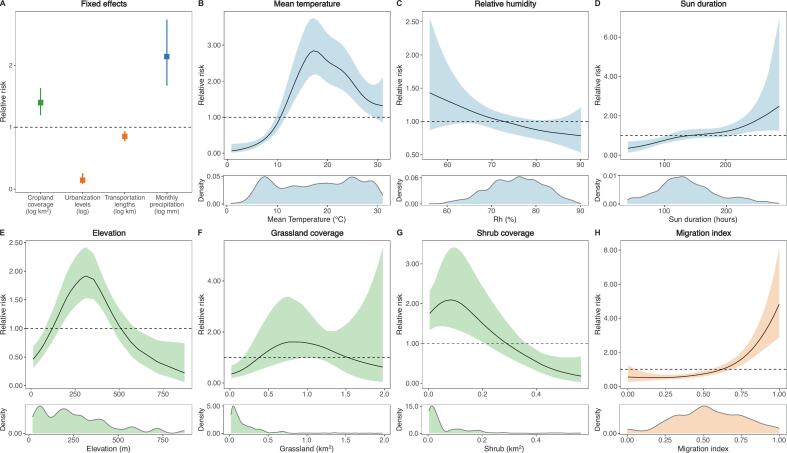
Effects of geographical, climatic, and social factors on SFTS incidence in Zhejiang, China, from 2011 to 2023. The sub-panels display posterior marginal linear fixed effects and nonlinear effects from the full model of county-level SFTS incidence. Fixed effects are presented as relative risks, with points and error bars indicating the posterior marginal mean and the corresponding 95% credible intervals. The nonlinear marginal effects are specified using a second-order random walk (see Methods for details) and shown as relative risk, where lines and bands indicate posterior means and their 95% credible intervals. The bottom panels show the density distribution of covariates. The final model incorporated the lagged and cumulative effects of climatic covariates, including average temperature, lagged 3-month relative humidity, 1-month cumulative sunshine duration, and 2-month cumulative monthly precipitation.

Environmental, climatic, and social factors are all associated with SFTS disease burden in Zhejiang (Fig. 2). Elevation exhibited a nonlinear association with SFTS risk, characterized by an inverted U-shaped pattern within the observed elevation range (121–503 m), with risk increasing at lower elevations and declining after reaching a peak at approximately mid-range elevations (around 300 m); elevations outside this range were associated with lower risk. Higher SFTS risk was associated with low shrub, and high cropland coverage. In contrast, high shrub, and low grassland coverage were linked to lower risk. Subgroup analyses revealed regional heterogeneity in environmental risk factors, suggesting that their influence on SFTS transmission may be context-dependent and shaped by environmental features that have not been explicitly described in our models (Fig. S11). But the subgroup analyses should be interpreted cautiously, as stratification substantially reduced the number of observations within each regional group and likely limited statistical power, particularly given the relatively low incidence of SFTS.

Among climatic factors, the association between mean temperature and SFTS risk was nonlinear, with risk increasing at lower temperatures and declining after reaching a peak at intermediate levels (around 18 °C). Sunshine duration also showed a nonlinear positive association, with the effect becoming more pronounced at higher levels (approximately above 200 h per month). Precipitation was associated with increased SFTS risk, whereas relative humidity showed no significant association. Among social factors, urbanicity and highway lengths per capita were both negatively associated with SFTS risks. While urbanicity is negatively associated with SFTS burden, cases reported in Zhejiang's mega-urban centre have increased quickly (Fig. S12). Human mobility was positively associated with SFTS risk, with higher migration index (>0.64) corresponding to increased risk, while lower mobility levels were associated with reduced risk.

**Fig. 3:**
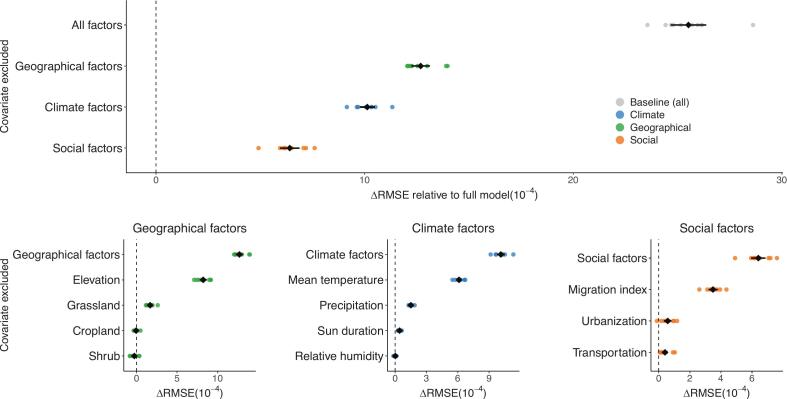
Individual influence of geographical, climatic, and social factors on spatiotemporal predictions of SFTS incidence. Using 5-fold spatiotemporal block cross-validation, the impact of each covariate on the out-of-sample root mean square error (RMSE) was evaluated by individually removing specific covariates from the full model. The y-axis illustrates the candidate models, which consist of models with one specific covariate removed each time from the full model, and the baseline model with all covariates excluded. Larger ΔRMSE indicates larger individual influence of the specific covariate. Each circle point represents the change in RMSE of the candidate models compared to the full model in each cross-validation experiment. This experiment was repeated 10 times. Black dots and error bars represent the mean and 95% confidence intervals of all 10 repetitions.

Compared to the null model (with no additional risk factors), the full model reduced the prediction error by ΔRMSE = 0.0026 (95% CI, 0.0025–0.0027) (Fig. 3) and explained 71.1% of the total variance compared (Fig. S13). Environmental, climatic, and social factors explained 38.5%, 23.2% and 9.4% of the model variance, respectively (Fig. 3, Fig. S14). Within these risk factor categories, elevation (ΔRMSE = 0.0008), mean temperature (ΔRMSE = 0.00061), and mobility (ΔRMSE = 0.00035) contributed the most to explaining the variance in the data. These three factors also exhibited the strongest effect sizes in univariate regression.

As a sensitivity analysis, the zero-inflated negative binomial model yielded consistent results with those of the primary negative binomial model, indicating that potential zero inflation did not materially influence the results or alter the main conclusions (Table S3, Fig. S14).

**Fig. 4:**
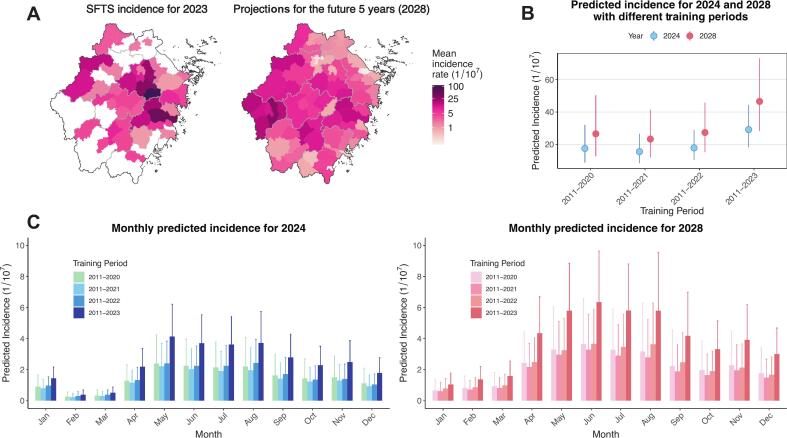
Projections of future SFTS burden and distribution with uncertainty analysis using different training sets. (A) Comparison between observed SFTS incidence in 2023 and projected SFTS incidence in 2028 (training window 2011–2023). The maps show the mean incidence rate per 10^7^ population, with a color gradient from light pink (lower incidence) to dark purple (higher incidence). (B) Predicted annual mean SFTS incidence for 2024 and 2028 using different training periods. This panel evaluates the impact of varying training datasets on projections, incorporating uncertainty in the estimates. (C) Monthly predicted SFTS incidence for 2024 and 2028 based on different training periods. This panel illustrates seasonal variation in future incidence and the influence of different historical data lengths on prediction outcomes.

**Fig. 5:**
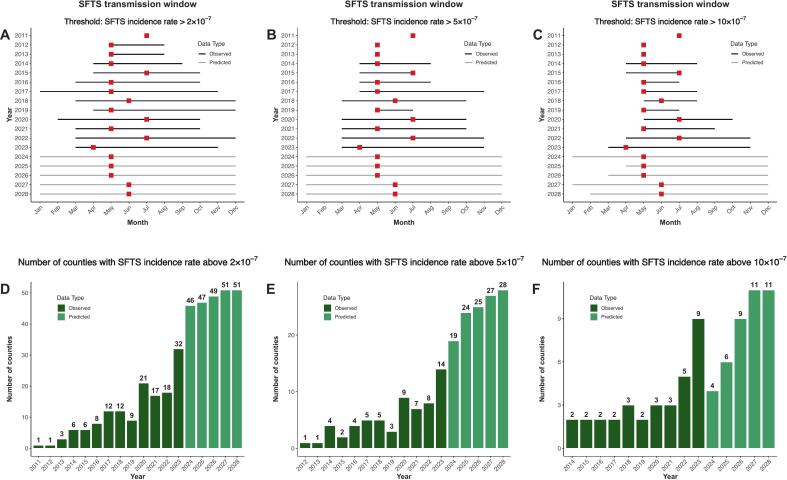
Transmission window and spatial spread pattern of SFTS in Zhejiang, China (2011–2028). Panels A–F show the observed and predicted SFTS transmission windows (A, B, C) and the number of affected counties (D, E, F) under three epidemic thresholds (2, 5, 10 cases per 100 million person-years). The squares represent peak month for SFTS, while the error bars represent the range of high-prevalence months exceeding the defined threshold. Observed and predicted values are based on surveillance data and model estimates using the training period 2011–2023, respectively.

With the current trajectory of climate change, SFTS will likely spread to new regions by 2028. Eight counties (of 43) that have never reported cases are expected to record at least one case (Fig. 4A, Fig. 5B, D, F). Further, the SFTS burden is expected to increase between 2023 and 2028 (Fig. 4B). Using the model trained using data between 2011 and 2023, we estimated that the mean incidence rate of SFTS in 2028 is 46.6 [95%CI: 28.3–73.1] per 10^7^ person-years, compared to 37.8 in 2023 and 7.1 in 2019. This translates to 309 cases per year in 2028, compared to 251 cases in 2023 and 45 cases in 2019, representing a 23% increase from 2023 (rate ratio: 1.23 [95% CI, 1.12–1.31]) and a 560% increase from 2019 (rate ratio: 6.60 [95% CI, 3.97–10.42]). Sensitivity analysis using models fitted on truncated time series led to similar interpretations and conclusions despite predicting a lower burden (Fig. 4B, C). Sensitivity analysis using proportionally adjusted future climate projections yielded similar conclusions (Fig. S15).

Between 2011 and 2023, SFTS seasons expanded significantly in Zhejiang regardless of the outbreak threshold we applied (Fig. 5A, B, C). Public health practitioners used to only expect SFTS cases in the spring-summer range – now they have to expect them in the spring-autumn range. Looking into the future, SFTS seasons are likely to further expand and may be reported all year round in some counties of Zhejiang by 2028, even under strict outbreak definitions (≥10 cases per 10^7^ population). Sensitivity analysis using forecasting models trained without 2023 data led to similar conclusions (Fig. S16). Similar patterns were observed when epidemic season was defined using cumulative case distribution–based criteria (>70%, >80%, and > 90% of annual cases), further supporting the robustness of our findings (Fig. S17).

The number of counties reporting outbreaks has increased substantially. Between 2011 and 2016, only 2–4 counties reported outbreaks each year. This number rose to 5–20 counties annually during 2019–2023 and is projected to reach 9–49 counties per year between 2024 and 2028 (Fig. 5D–F).

## 4 Discussion

Using data from 2011 to 2023, we showed that environmental, climatic, and social risk factors are associated with SFTS disease burden. Environmental risk factors contributed the most to explaining the variability observed in SFTS disease burden, followed by climatic and social risk factors. Within each risk factor category, the most influential variables are elevation, temperature, and human mobility, respectively. In the context of global climate change, Zhejiang is expected to face a greater SFTS risk by 2028. More of Zhejiang's counties will likely be impacted. As a disease that only affected Zhejiang's population for less than five months of the year between 2011 and 2023, SFTS may be reported most of the year soon. SFTS surveillance, prevention, and control measures urgently need to be incorporated into routine public health management.

Previous studies have explored the relationship between geographical and climatic factors and SFTS risk [14], [15], [16], [50]. Consistent with prior research, our results show a nonlinear, U-shaped association between elevation and SFTS incidence, with risk increasing at mid-elevations and decreasing at higher altitudes [16], [38]. This pattern may reflect ecological and behavioral mechanisms, as moderate elevations often support suitable vegetation and host habitats while maintaining active human–animal interactions. In contrast, high-altitude regions may limit both tick survival and host activity [16], [38]. We also identified a positive association between cropland coverage and SFTS risk in Zhejiang. This finding aligns with existing evidence that agricultural workers constitute a high-risk population due to frequent occupational exposure to ticks during farming activities [51], [52]. In addition, cropland environments may provide favorable habitats for SFTSV reservoir hosts, including rodents and livestock, thereby facilitating vector–host contact and viral maintenance [51], [52]. The associations with grassland and shrubland coverage were more complex and less consistent with findings from other endemic regions [52]. In provinces where grassland and shrubland are more extensive, these landscapes have been linked to increased SFTS risk. In contrast, their relatively limited distribution in Zhejiang may attenuate their influence on local transmission dynamics. These findings highlight the context-dependent nature of environmental risk factors and suggest that the ecological determinants of SFTS transmission may vary across regions according to landscape composition and human land-use patterns [53]. However, the coverage of these two types of terrain in Zhejiang is both low – our results thus should be interpreted with caution.

Regionally stratified analyses further showed that the associations between environmental factors and SFTS vary drastically across Zhejiang. The links between risk factors and SFTS risk may be specific to the local context. This is also observed for other climate-sensitive infectious diseases such as dengue fever. This regional heterogeneity highlights the importance of local ecological contexts in shaping disease risk and reinforces the need for region-specific assessments [53].

The association between temperature and SFTS risk was nonlinear, with the highest risk observed at intermediate temperature levels (around 18 °C). Warmer temperatures may increase the risk of tick-borne diseases by promoting tick development and activity, while also increasing human outdoor exposure [54], [55]. Precipitation and sunshine duration were also associated with increased SFTS risk, consistent with previous studies [15], [36]. Increased precipitation can enhance vegetation density and soil moisture, creating favorable microhabitats for ticks and their animal hosts, and supporting the survival of immature tick stages. A longer duration of sunshine may further increase host activity (e.g., grazing behavior) and extend human outdoor exposure time, thereby increasing opportunities for contact with infected ticks [15], [16], [36], [55]. In contrast, the association between humidity and SFTS risk has been less consistent [15], [36], [56]. In our study, no independent association was observed after adjustment for other climatic factors. Although higher humidity may reduce tick desiccation, its effect may be moderated by interactions with other environmental variables.

Compared to environmental and climatic factors, social factors have received less attention in the context of SFTS [44], [48], although their impacts may be important and complex [57], [58]. In this study, we found that mobility is strongly associated with SFTS risks. Among the social indicators examined, the migration index showed the strongest association with SFTS incidence. This hypothesis-generating finding suggests that human mobility may contribute to shaping the spatial distribution of SFTS risk, although it does not imply that human-to-human transmission is the dominant pathway. We have only found one other study that explored the role of human mobility using data from the COVID-19 pandemic period [30]. Given the differences in epidemiology, study period, underlying population, and metrics that measure mobility, we cannot compare our results directly. Mechanistically, human mobility may influence SFTS transmission by altering patterns of population exposure across regions [59], [60]. Population movement can increase the likelihood of contact with infected ticks in ecologically suitable areas, particularly when individuals travel between high-endemic and low-incidence regions. This may contribute to the spatial redistribution of cases and increase the risk of introduction into previously unaffected areas [59], [60].

Urbanicity and highway lengths per capita are negatively associated with SFTS risks. Human activities and transportation infrastructure may disrupt the natural habitat of ticks and may reduce direct exposure to tick-presence environments for local inhabitants [54]. From a mechanistic perspective, urbanization may reduce SFTS risk by transforming land cover and decreasing the availability of suitable habitats for ticks and their animal hosts. Highly urbanized areas are typically characterized by reduced vegetation cover and lower densities of wildlife hosts, which may limit vector survival and disrupt transmission cycles [18]. They may also be proxies for socioeconomic status, including for the access and quality of healthcare and public health services. Public health campaigns against SFTS may be more effective where infrastructure is better developed [48]. However, the negative association between urbanicity and SFTS risks may not persist as urban green spaces emerge as new tick habitats [25], [26]. In China, South Korea, and Thailand, SFTSV transmission has occurred through ticks found in such urban green spaces.

While our analysis primarily focused on macro-level social indicators (e.g., urbanization, mobility intensity, transportation infrastructure), a substantial body of evidence demonstrates that individual-level social risk factors (occupation, behavioral habits, age) play a pivotal role in determining both infection risk and clinical outcomes of SFTS [61], [62]. Epidemiological surveillance data consistently show that agricultural workers account for a large proportion of reported cases, frequently exceeding 70–90% in endemic regions of China and neighboring countries [19], [61], [63] This occupational concentration reflects prolonged exposure to tick habitats during farming, tea picking, livestock management, and other outdoor activities [63]. Age is another critical determinant. SFTS cases are disproportionately concentrated among middle-aged and older adults, and advanced age has repeatedly been identified as a strong predictor of mortality [19], [64]. In addition, specific outdoor behaviors—such as resting directly on grassland, inadequate use of personal protective equipment, and close contact with livestock or companion animals—have been identified as independent risk factors for infection [61], [62]. Together, these findings suggest that macro-level drivers such as mobility and urbanization provide the structural conditions for spatial spread, whereas occupation, age, and behavioral exposures determine individual vulnerability within affected regions. Future research integrating individual-level exposure data with macro-scale spatial models may further enhance risk stratification and targeted intervention strategies.

SFTS has historically been considered a rare infectious disease in Zhejiang. There has been an upward trajectory in disease burden between 2011 and 2023. Based on model projections, we expect the SFTS burden to continue increasing by 2028 in the context of global climate change. More counties will be affected – more healthcare workers and public health practitioners who may never have seen SFTS infections may have their first encounter soon. Further, seasons during which healthcare workers and public health practitioners may need to be particularly careful with SFTSV transmission are expanding from 4 to 5 months to 10–12 months. This shift from a historically concentrated spring–summer pattern toward a prolonged transmission period has important implications for surveillance planning. Public health systems that traditionally operate enhanced monitoring during a limited seasonal window may need to consider extending preparedness efforts throughout the year. Similarly, maintaining clinical awareness and diagnostic readiness beyond peak months may reduce delayed detection in newly affected areas.

It is also important to acknowledge that the observed increase in reported SFTS cases over time may partly reflect enhanced surveillance sensitivity, improved diagnostic capacity, and heightened public and clinician awareness of the disease [65]. As SFTS surveillance primarily relies on passive case detection through healthcare-seeking behavior followed by laboratory confirmation, improvements in case recognition and reporting practices may contribute to temporal increases in notified incidence [66]. Therefore, the upward trend in reported cases likely reflects a combination of true transmission dynamics and evolving surveillance systems. Future studies incorporating active surveillance data or seroprevalence surveys may help disentangle true transmission changes from reporting effects.

In addition to seasonal expansion, we project that approximately 9% additional counties may report their first SFTS cases by 2028. This geographic expansion suggests that counties without prior reported cases could benefit from early capacity-building, including clinician training, laboratory preparedness, and risk communication strategies. Strengthening surveillance in ecologically transitional zones may facilitate earlier identification of emerging hotspots. These changes necessitate greater public health capacity than is currently in place. Training to establish awareness of SFTS epidemiology will be needed in many more counties. The surveillance system may need to be improved to achieve greater detection power and longer active surveillance periods. Additionally, more effective interventions, such as public health information campaigns and vector control measures, may help to mitigate the upward trajectory of future SFTS disease burden [14].

In infectious disease epidemiology, spatial autoregressive terms are often included in statistical modelling exercises because locations closer to each other may have similar experiences. However, in this study, after adjusting for environmental, climatic, and social risk factors, spatial autoregressive terms do not introduce additional predictive power. This suggests that spatial dependencies were either irrelevant or already captured by other covariates. The finding that mobility explains a measurable proportion of the variance in SFTS incidence and diminishes the added value of spatial autoregressive terms suggests that mobility may capture latent spatial transmission processes. Incorporating real-time migration indicators or transportation metrics into surveillance systems may enhance spatial risk assessment and provide an additional layer of early warning, particularly in rapidly urbanizing regions. Future research should focus on uncovering the specific underlying mechanism.

To our knowledge, relatively few studies have comprehensively examined social risk factors in SFTS transmission, and our findings add further evidence to this area. We relied on a rigorous and data-driven model development process that involved various functional forms of the independent variables, various model diagnostics metrics, and multiple assumptions around spatial and temporal dependency structures. Because the functional relationships between environmental, climatic, and social drivers and SFTS risk are not well established, we did not impose linear assumptions a priori. For future projection, to avoid over-fitting, we applied multiple truncations to the time series that we were working with. In terms of result interpretation, we took a public health management lens. Because SFTS remains a relatively rare emerging disease without a standardized outbreak definition, we evaluated multiple incidence thresholds rather than adopting a single cutoff, allowing seasonal transmission patterns to be interpreted across different levels of transmission intensity. Although we do not commit strongly to these outbreak thresholds, our findings suggest a broader yet consistent conclusion: the seasonal window for SFTS transmission has expanded, placing additional pressure on already at-capacity healthcare and public health systems. Leveraging multiple context-specific outbreak thresholds can provide a more nuanced understanding of emerging vector-borne diseases and should be promoted in future research.

Some limitations are worth noting. First, reporting or ascertainment issues may exist, although Zhejiang is considered to have strong infectious disease surveillance systems. Non-specific symptoms of SFTS, heterogeneity in healthcare-seeking behaviors, and the lack of clinical experience among healthcare workers may all contribute to this. Subject-specific training among healthcare workers and public health practitioners may help address this issue. Second, while SFTS is a tick-borne zoonotic disease, we do not currently have access to systemic surveillance data on SFTS virus detection in ticks or in farm, domesticated or wild animals, even though sporadic testing efforts exist. Such data will undoubtedly improve the prediction power of our model. Thirdly, an important limitation of this study is the absence of direct tick density measurements. Although tick abundance plays a central role in SFTS transmission dynamics, currently available surveillance data are incomplete and not standardized across regions and years. To partially address this limitation, we incorporated multiple environmental and climatic variables—such as elevation, land cover, temperature, and precipitation—that are strongly associated with tick habitat suitability and seasonal activity [14], [67]. These ecological proxies may capture part of the spatial and temporal variation in tick density [14], [67]. However, future studies integrating systematic vector surveillance data would further strengthen mechanistic inference. Lastly, we may not have fully explored the uncertainty around the future projection of climatic factors despite sensitivity analysis by altering the time-series lengths used. Although population-related and socioeconomic factors (e.g., urbanicity and mobility) were incorporated into projections through historical trend extrapolation, this approach assumes continuity of past trends and does not explicitly model structural demographic or economic transitions. Deviations from these trends may introduce uncertainty into the projected SFTS burden.

## 5 Conclusions

The burden of SFTS in Zhejiang, a tick-borne zoonotic infectious disease with a high case fatality ratio, is associated with environmental, climatic, and social risk factors. Elevation, mean temperature, and human mobility are leading factors affecting SFTS risks. In the context of global climate change, Zhejiang should expect a large increase in SFTS burden by 2028, accompanied by geographic expansion of epidemic regions. SFTS seasons will also become longer than the current ones. These projected changes underscore the importance of adapting surveillance systems, strengthening clinician preparedness in newly affected areas, and considering mobility-informed monitoring strategies. A flexible, multi-threshold framework for defining outbreaks may further support tailored public health responses in regions with varying baseline risks.

## Authors contribution

**Yating Yang**: conceptualization, methodology, software, data curation, formal analysis, writing – original draft, writing – review and editing, visualization. **Yang Liu**: conceptualization, methodology, writing – original draft, writing – review and editing, visualization. **Ye Yao**: conceptualization, methodology, acquisition and interpretation of data, writing – review and editing, visualization. **Jimin Sun**: conceptualization, data curation, acquisition and interpretation of data, writing – review and editing, obtained funding. **Ying Liu**: conceptualization, data curation, acquisition and interpretation of data, writing – review and editing, obtained funding. **Jiangping Ren**: data curation, writing – review and editing, obtained funding. **Guoyou Qin**: methodology, writing – review and editing; **Yihan Lu**: conceptualization, methodology, writing – review and editing.

## Declaration of generative AI in scientific writing

ChatGPT-3.5 and 4o have been used to correct for grammar and to improve the readability of this manuscript.

## CRediT authorship contribution statement

**Yating Yang:** Writing – review & editing, Writing – original draft, Visualization, Validation, Software, Methodology, Formal analysis, Conceptualization. **Jiangping Ren:** Writing – review & editing, Funding acquisition, Data curation. **Guoyou Qin:** Writing – review & editing, Methodology. **Yihan Lu:** Writing – review & editing, Methodology. **Ying Liu:** Writing – review & editing, Supervision, Funding acquisition, Data curation, Conceptualization. **Jimin Sun:** Writing – review & editing, Supervision, Funding acquisition, Data curation, Conceptualization. **Ye Yao:** Writing – review & editing, Visualization, Validation, Supervision, Methodology, Data curation, Conceptualization. **Yang Liu:** Writing – review & editing, Writing – original draft, Visualization, Validation, Supervision, Methodology, Investigation, Conceptualization.

## Funding/support

This study was supported by grants from Zhejiang Provincial Science and Technology Program for Disease Prevention and Control (2025JK186, 2025JK163), Zhejiang Province Vanguard Goose-Leading Initiative (2024C03216, 2025C02186), the Key Program of Health Commission of Zhejiang Province/Science Foundation of National Health Commission (WKJ-ZJ-2523), the National Key Research and Development Project by the Ministry of Science and the Technology of China (2023YFC2308705), and Key projects of the 10.13039/501100001809National Natural Science Foundation of China (U23A20496) to Jiming Sun, Ying Liu and Jiangping Ren. The funders had no role in study design, data collection and analysis, decision to publish, or preparation of the manuscript.

## Role of the funder/sponsor

The funders had no role in the design and conduct of the study; collection, management, analysis, or interpretation of the data; preparation, review, or approval of the manuscript; and decision to submit the manuscript for publication.

## Declaration of competing interest

The authors declare no conflict of interest.

Ethics approval and consent to participate.

*The study was approved by the Institutional Review Board of the Centres for Disease Control and Prevention in Zhejiang (IRB#2025–017-01). Informed consent from participants involved in the study was waived since we only used anonymised data exported from surveillance systems.

## Data Availability

Data will be made available on request.

## Appendix A Supplementary data

Supplementary material Image 1
