# Supplementary material for: Environmental, climatic, and social risk factors of severe fever with thrombocytopenia syndrome and the implications of climate change
Source: One Health. 2026 Apr 26;22:101430. doi: 10.1016/j.onehlt.2026.101430 (PMC13141556; doi:10.1016/j.onehlt.2026.101430)
Supplement: Supplementary file 1 — Supplementary material [file mmc1.docx]

Supplementary Information for:

**Environmental, Climatic, and Social Risk Factors of Severe Fever with Thrombocytopenia Syndrome and the Implications of Climate Change**

Yating Yang^1^, Jiangping Ren^2^, Guoyou Qin^1,4^, Yihan Lu^3,4^, Ying Liu^2^*, Jimin Sun^2^*, Ye Yao^1,4^*#, Yang Liu^5,6, 7^#

**Affiliations**

^1^ Department of Biostatistics, National Commission of Health Key Laboratory for Health Technology Assessment, and the Key Laboratory of Public Health Safety of Ministry of Education, School of Public Health, Fudan University, Shanghai, China.

^2^ Zhejiang Key Lab of Vaccine, Infectious Disease Prevention and Control, Zhejiang Provincial Center for Disease Control and Prevention, Hangzhou, China

^3^ Department of Epidemiology, Ministry of Education Key Laboratory of Public Health Safety, School of Public Health, Fudan University, Shanghai, China.

^4^ Shanghai Institute of Infectious Disease and Biosecurity, Fudan University, Shanghai, China.

^5^ Centre for Mathematical Modelling of Infectious Diseases, London School of Hygiene & Tropical Medicine, London, United Kingdom.

^6^ Centre on Climate Change and Planetary Health, London School of Hygiene & Tropical Medicine, London, United Kingdom.

^7^ Department of Infectious Disease Epidemiology and Dynamics, Faculty of Epidemiology and Population Health, London School of Hygiene & Tropical Medicine, London, United Kingdom.

# These authors contributed equally.

**^*^Correspondence:** Ye Yao, 130 Dong’an Road, Shanghai, 200032, China. Email: [yyao@fudan.edu.cn](mailto:yyao@fudan.edu.cn);

Jimin Sun, 3399 Binsheng Road, Hangzhou, 310051, China. Email: [jmsun@cdc.zj.cn](mailto:jmsun@cdc.zj.cn);

Ying Liu, 3399 Binsheng Road, Hangzhou, 310051, China. Email: [yliu@cdc.zj.cn](mailto:yliu@cdc.zj.cn).

**Supplementary Materials**

**Supplementary Methods**

1. Data collection

1.1 The inclusion criteria of SFTS

1.2 The details of data cleaning on future climate conditions

1.3 The details of data sources and transformations for covariates.

2. Model development

2.1 Data processing and variable selection

2.2 Addressing multicollinearity

2.3 Model structure and spatial-temporal structure assumption

2.4 Validation

3. Statistical analyses

3.1 Measuring the predictive influence of covariates via block cross-validation

3.2 The calculation for the variance explained by each type of variable

4. Sensitivity Analysis

4.1 Using Zero-Inflated Negative Binomial Model

4.2. Sensitivity analysis for projecting SFTS burden under future socio-environmental and climatic changes

4.3. Sensitivity analysis of the transmission window and spatial spread pattern of SFTS in Zhejiang, China using forecasting models trained without 2023 data

4.4 Sensitivity Analysis for Temporal Interpolation

4.5 Sensitivity Analysis Using Alternative Season Definitions

**Supplementary Figures & Tables**

Table S1. Data sources of SFTS and its potential drivers.

Figure S1. Flowchart of model construction

Figure S2. Temporal trends of SFTS incidence across cities and counties in Zhejiang, China.

Figure S3. Annual trend of geographic variables.

Figure S4. The lagged and cumulative effects of climate variables within six months

Figure S5. Selecting the best-fitting type for each covariate

Figure S6. Correlation matrix for each pair of variables

Figure S7. Variance inflation factor (VIF) for each variable.

Table S2. Summary of Tested Models with Varying Spatiotemporal Structures

Figure S8. Validation results for model with varying spatiotemporal structures: case 1

Figure S9. Validation results for model with varying spatiotemporal structures: case 2

Figure S10. Validation results for model with varying spatiotemporal structures: case 3

Figure S11. Effects of socio-environmental, geographical, and climatic drivers on SFTS incidence stratified by regions

Figure S12. Proportion of top 10 counties with highest SFTS cases

Figure S13. Proportion of variance explained by each type of variables

Table S3. Comparison of Model Fit Statistics Between Negative Binomial (NB) and Zero-Inflated Negative Binomial (ZINB) Models

Figure S14. Effect Estimates from the Zero-Inflated Negative Binomial (ZINB) Model

Figure S15. Sensitivity analysis for projecting SFTS burden under future socio-environmental and climatic changes

Figure S16. Sensitivity analysis of the transmission window and spatial spread pattern of SFTS in Zhejiang, China using forecasting models trained without 2023 data

Table S4. Sensitivity Analysis Comparing Monthly Expansion and Spline-Interpolated Annual Covariates

Figure S17. Sensitivity analysis of SFTS epidemic season definitions using cumulative case distribution–based criteria

**Supplementary Text 1. Data collection**

**1.1 The inclusion criteria of SFTS**

According to the guidelines for the prevention and treatment of sever fever with thrombocytopenia syndrome (SFTS) issued by the Ministry of Health of the People's Republic of China in 2010^1^, all SFTS cases diagnosed at any level of medical institution must be reported to the China Information System for Disease Control and Prevention (CISDCP) within 24 hours of diagnosis. Reported cases are defined according to the national guidelines for the diagnosis and treatment of SFTS:

1. Suspected cases are defined as those with any of the following epidemiological histories, along with corresponding clinical manifestations:
2. History of working, living, or traveling in hilly, forested, or mountainous areas during the endemic season.
3. History of tick bites within two weeks before the onset of symptoms.
4. Contact history with infected animals or confirmed cases.
5. Clinically diagnosed cases are defined as suspected cases with any of the following:
6. Positive SFTSV-IgM.
7. Manifestations of multi-organ dysfunction.
8. Confirmed cases are defined as suspected or clinically diagnosed cases with any of the following:
9. Positive SFTSV nucleic acid.
10. Isolation of SFTSV from clinical specimens.
11. SFTSV-IgG seroconversion or a fourfold or greater increase in titers during the convalescent phase compared to the acute phase.

We collected data on all SFTS cases in Zhejiang Province from January 1, 2011 to December 31, 2023 through the CISDCP system and summarized the monthly case numbers for each county-level administrative region for analysis. Cases from offshore islands were excluded due to their distinct climate, ecological conditions, and geographic separation from the mainland^2,3^.

**1.2 The details of data cleaning on future climate conditions**

A high-resolution monthly temperature dataset over China was utilized in this study, derived from the Coupled Model Intercomparison Project Phase 6 (CMIP6). The dataset covers the period from January 2021 to December 2100 and has a spatial resolution of approximately 1 km (0.0083333°). It was generated using a Delta spatial downscaling approach applied to the global CMIP6 climate model outputs (>100 km resolution) and the WorldClim high-resolution climate dataset^4^.

The dataset incorporates three Shared Socioeconomic Pathway (SSP) scenarios (SSP119, SSP245, and SSP585), each including three General Circulation Models (GCMs): EC-Earth3, GFDL-ESM4, and MRI-ESM2-0. The geographic coverage is limited to mainland China, excluding regions such as the South China Sea islands. Temperature values are recorded in units of 0.1°C.

For this study, we first screened the three GCMs provided in the dataset. Based on a comparison of historical simulations with observed temperature data over China, the GCM with the smallest bias was selected for analysis. This ensures that the model used aligns most closely with observed climate conditions, thereby improving the reliability of future projections. For detailed information on the data processing and downscaling methodology, please refer to the original dataset documentation and related literature^4^.

In this study, we applied the Delta method to correct the bias in future climate projection data^5^. The Delta method adjusts future climate model outputs based on the differences between historical simulations and observations. For our research, the future climate dataset encompasses data from 2021 to 2100, and we utilized the historical data from 2021–2023 to adjust the simulated data for the period 2024–2028. We calculated the mean difference or ratio of means between the model-simulated historical data and the observed data during the baseline period. These correction factors were then applied to the future climate model projections to obtain bias-corrected data. Specifically, the mean difference (for temperature) or the ratio of means (for precipitation) between the model-simulated historical data and the observed data during the baseline period (2021–2023) was calculated and applied to the future projections (2024–2028). The Delta method assumes that the relative bias in the model's simulation remains consistent over time, allowing for the adjustment of future projections using the determined correction factors from the historical period. It's important to note that while the Delta method corrects the mean bias, it does not account for changes in the distribution shape or variability of the data. However, its simplicity and effectiveness in many cases make it a valuable tool for bias correction in climate studies.

**1.3. The details of data sources and transformations for covariates.**

**Climate data:** Monthly climate data for each municipal district from 2011 to 2023, including average temperature, highest temperature, lowest temperature, relative humidity, cumulative precipitation, and sunshine duration, were obtained from the China Meteorological Data Service Center (<http://data.cma.cn>).

**Land Cover Data:** Data were sourced from the annual China Land Cover Dataset (CLCD), ^6^, which was generated using Landsat satellite data on the Google Earth Engine (GEE) platform. This dataset provides 30-meter resolution land cover data for China from 1990 to 2022, encompassing nine land use types: cropland, forest, shrubland, grassland, water bodies, ice and snow, bare land, impervious surfaces, and wetlands. Given that SFTS is prevalent in mountainous and hilly areas, we calculated the area (km^2^) of cropland, forest, shrubland, and grassland as candidate variables. Data were summarized to county-level for analyses.

**Topography data:** Data were obtained from the Copernicus Digital Elevation Model (DEM) dataset, which offers a 30-meter resolution and is widely recognized as the most reliable global open-source DEM, released by the European Space Agency ^7^. Data were aggregated at the county level for analysis.

**Urbanization:** The urbanization level is measured by the proportion of the population residing in urban areas, based on data from the Zhejiang Statistical Yearbook.

**Transportation length:** The data on per capita transportation length was obtained from the Zhejiang Statistical Yearbook. It is defined as the total length of urban highway operating lines. Notably, Wenzhou experienced a significant increase in transportation length in 2017, largely due to previous challenges with limited travel options and scarce highways. To address these issues, Wenzhou introduced policies that prioritized transportation development, leading to the construction of several major highways. Between 2015 and 2017, Wenzhou successfully connected all counties via highways, greatly improving travel conditions for rural residents and elevating its total transportation length from the third lowest to one of the top three in the province.^8^

**Population migration scale:** The Baidu migration scale index was derived from Baidu migration data (<https://qianxi.baidu.com/>). Baidu migration data is sourced from the vast location-based service (LBS) data provided by the Baidu Map Open Platform. This data comes from all software that uses Baidu's location-based API services. According to official reports, the Baidu Map Open Platform is the preferred choice for many developers. There are over 1.65 million developers using the platform, which holds a 75% market share and serves more than 650,000 active apps and websites. From a user perspective, Baidu’s location services respond to over 120 billion global location requests daily. This extensive data forms the foundation of the migration data. To eliminate bias due to the wide range of values in the Baidu migration scale across different cities, we standardized the index, scaling it to a 0-1 range as follow:

$$z = \frac{x-min(x)}{max(x)-min(x)}$$

The missing data were imputed using the random forest multiple imputation algorithm, performed by *mice* package in R version 4.4.0.

A comprehensive literature review was conducted to identify potential geographical, climatic, and socio-environmental covariates associated with SFTS. The ecological rationale and supporting references for each variable are summarized in Table S1.

| Table S1. Data sources of SFTS and its potential drivers.^1^ | | | |  |
| --- | --- | --- | --- | --- |
| **Type** | **Variables** | **Temporal and spatial resolution** | **Source** | **Rationale ^2^** |
| **Disease** | SFTS cases | Temporal: 2011-2023 Daily (summarized to monthly data);  Spatial: individual-level address with sociodemographic information (summarized to county-level) | China Information System for Disease Control and Prevention | Not applicable. |
| **Geography** | Coverage of forest (km^2^) | Temporal: 2011-2022 Annually;  Spatial: 30*30 m^2^ (summarized to county-level) | The Annual China Land Cover Dataset (CLCD) (<https://doi.org/10.5281/zenodo.8176941>)^36^ | Forest areas provide habitats for ticks and their host animals, increasing the risk of human-tick encounters.^18,31^ |
|  | Coverage of cropland (km^2^) |  |  | Cropland may influence tick populations and their interactions with human and animal hosts.^18^ |
|  | Coverage of shrubland (km^2^) |  |  | Shrubland areas can serve as habitats for ticks and their hosts, affecting tick density and human exposure.^18^ |
|  | Coverage of grassland (km^2^) |  |  | Grassland can influence the habitat suitability for ticks and their host animals.^32^ |
|  | Elevation (km) |  | Copernicus Digital Elevation Model (<https://panda.copernicus.eu/panda>) | Elevation affects climate conditions, vegetation types, and tick habitat distribution, impacting SFTS occurrence.^18,31^ |
| **Climate** | Mean temperature (℃) | Temporal: 2011-2023 Monthly;  Spatial: city-level | China Meteorological Data Service Center (<http://data.cma.cn>) | Temperature affects the development of ticks and influences human outdoor activities, impacting the frequency of human-tick contact.^33-35^ |
|  | Cumulative precipitation (mm) |  |  | Precipitation may indirectly affect the occurrence of SFTS by influencing the habitat condition of ticks. ^33-35^ |
|  | Relative humidity (%) |  |  | High humidity environments may provide more suitable conditions for tick survival. ^33-35^ |
|  | Sun duration (hour) |  |  | Increased sunlight duration may lead to longer outdoor stays for host animals, such as goats, and affects human outdoor activities and the frequency of tick activity. ^33-35^ |
| **Socio-environmental** | **Urbanization:** Percentage of the total population living in urban areas (%) | Temporal: 2011-2023 Annually;  Spatial: city-level | Zhejiang Province’s Statistical Yearbook (<https://tjj.zj.gov.cn/>) | Urbanization can affect human behavior, land use patterns, and contact rates with ticks.^42^ |
|  | **Mobility:** Population migration index (reflects the size of population flows) | Temporal: 2019.1-2019.3, 2020.1-2023.12 Daily (interpolated and summarized to monthly values);  Spatial: city-level | Baidu Migration Dataset (<https://qianxi.baidu.com/>) | Population movement patterns can influence the spread of SFTS by facilitating the movement of infected individuals and ticks.^43,54^ |
|  | **Mobility:** Travel length of transportation routes per capita (km) | Temporal: 2011-2023 Annually;  Spatial: city-level | Zhejiang Province’s Statistical Yearbook (<https://tjj.zj.gov.cn/> ) | Transportation infrastructure can impact human mobility and the potential for SFTS spread through increased human-tick interactions.^43^ |
| ^1^ Detailed description of covariates is explained thoroughly in Supplementary Text 2  ^2^ The reasons for including these factors are elaborated in the table, supported by an extensive literature review that identified potential variables influencing SFTS. However, the associations found in different studies are inconsistent. While these factors may be linked to SFTS, their exact relationship and the strength of their effects remain unclear. | | | | |

**Supplementary Text 2. Additional details in model development**
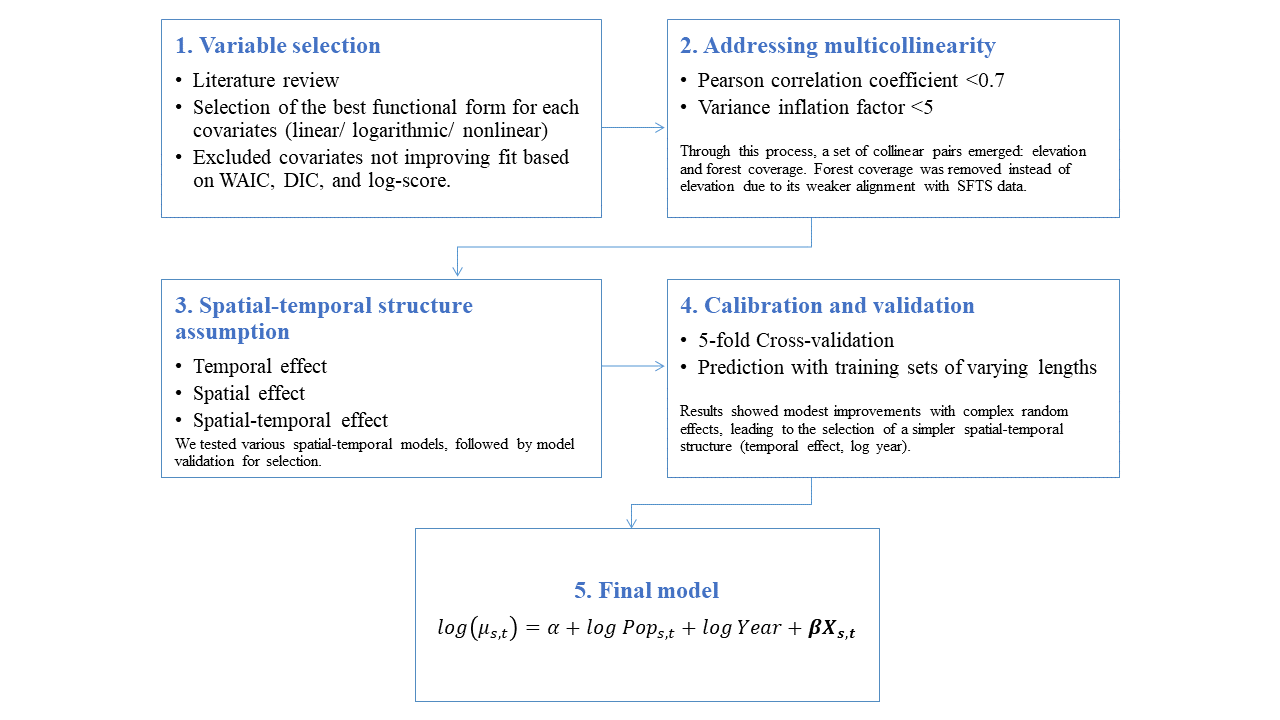


**Figure S1. Flowchart of model construction**

**2.1 Data processing and variable selection**

A comprehensive literature review was conducted to identify potential covariates associated with SFTS.^9-17^ This study considers three categories of covariates: geographical, climatic, and social factors, with the specific rationale for each variable detailed in Table 1. When prior knowledge regarding the relationship between covariates and the outcome is uncertain, systematically exploring different functional forms helps avoid unwarranted assumptions about these associations. According to the literature, the relationship between social factors and SFTS remains entirely unclear. While some studies suggest that social factors may influence vector-borne diseases, no research has specifically examined their role in SFTS transmission. Climatic factors have been extensively studied in relation to SFTS, but findings remain inconsistent. Most studies highlight temperature as an important determinant, yet the influence of precipitation, humidity, wind speed, and sunlight duration remain inconclusive. Geographical factors exhibit spatial heterogeneity, with their associations varying across different regions depending on local geographical conditions. To address these uncertainties, we adopted a data-driven approach to determine the most suitable functional form (linear, logarithmic, or nonlinear) for each covariate. The optimal transformation was selected based on the Deviance Information Criterion (DIC), with each functional form added individually to the baseline model that included only the logarithm of population density as an offset. A lower DIC indicated superior predictive performance (Figure S5).

Given the long life cycle of ticks, climatic factors may have prolonged effects on disease incidence by influencing tick reproduction. To account for these delayed impacts, we analyzed the lagged and cumulative effects of all climatic variables over a six-month period (Figure S4). The climatic variables with the strongest lagged or cumulative effects were included in the model.

Covariates that did not improve model fit, as evaluated using information criteria (WAIC, DIC, and log-score) in univariate models, were excluded. In this analysis, All variables were retained as they contributed to model prediction.

Since variable selection was data-driven, there is a potential risk of overfitting. Therefore, validation is essential to ensure the model’s reliability, which has been conducted in subsequent analyses.

Based on the above process, the covariates selected for the next step of analysis were: the nonlinear effects of elevation, nonlinear effects of forest area, nonlinear effects of grassland area, logarithm of cropland area, nonlinear effects of shrubland area, nonlinear effects of average monthly temperature, relative humidity, and sunshine duration, logarithm of monthly precipitation, logarithm of urbanization level, logarithm of transportation routes, nonlinear effects of in-migration index.

All variables were aligned to a county-month observational unit prior to modeling.

- **Outcome data:** Individual SFTS case records were aggregated to monthly counts at the county level.
- **Daily variables (e.g., mobility indices):** Daily observations were interpolated where necessary and summarized to monthly averages or totals to match the outcome scale.
- **Monthly climatic variables:** Mean temperature, cumulative precipitation, relative humidity, and sunshine duration were available at a monthly resolution and directly incorporated.
- **Annual geographical and socio-environmental variables:** Land-cover indicators (forest, cropland, shrubland, grassland), urbanization level, and transportation infrastructure were reported annually. These variables were expanded to monthly observations by assigning the annual value to all months within the corresponding year, under the assumption that structural environmental and socio-economic conditions change gradually within a calendar year.

This harmonization ensured that all covariates were temporally consistent prior to model estimation.

**Figure S2. Temporal trends of SFTS incidence across cities and counties in Zhejiang, China.**


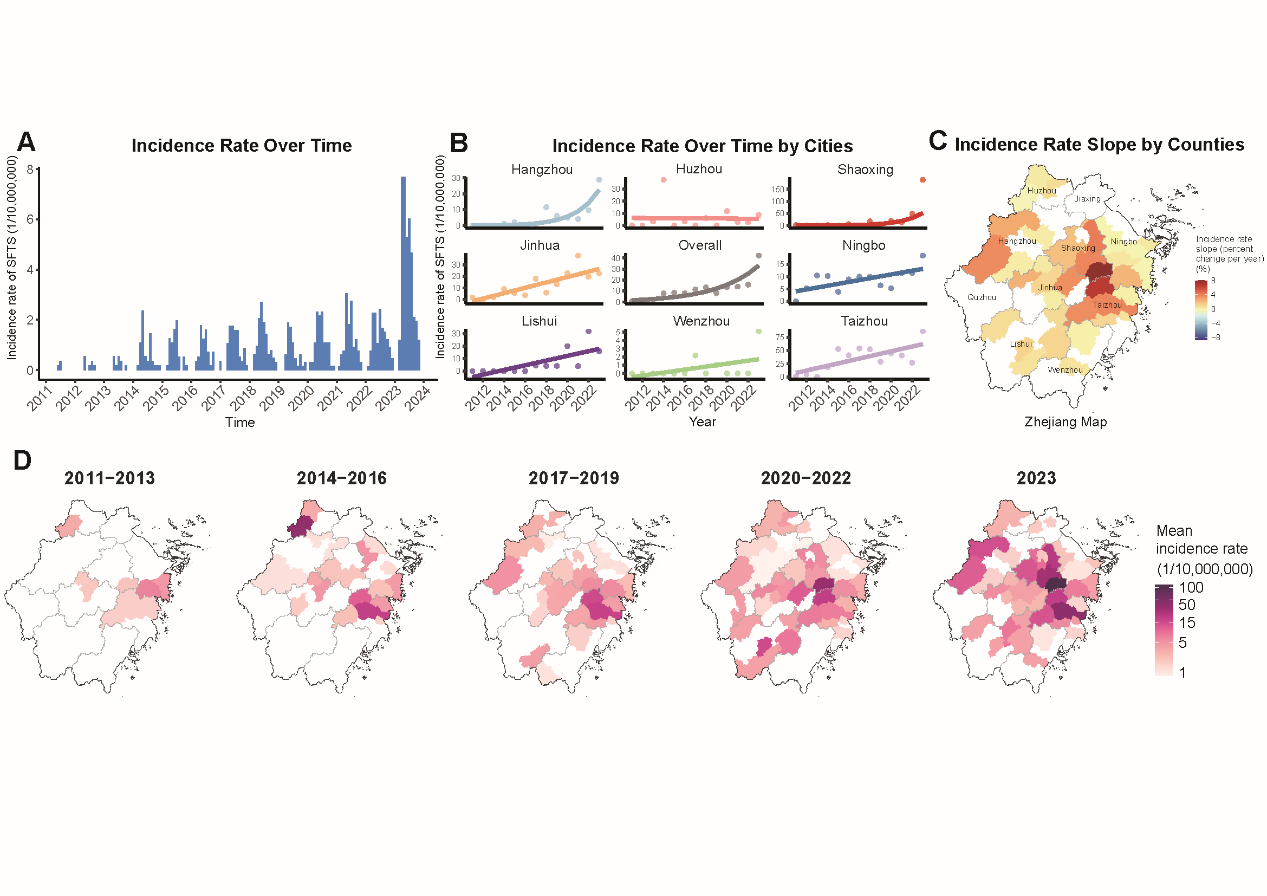


**(A)** Annual trend of SFTS incidence across the province from 2011 to 2023. The blue bars show the monthly average incidence rate of SFTS per 10,000,000 people. **(B)** Annual trends of SFTS incidence in different cities of Zhejiang Province from 2011 to 2023. Each subplot shows the incidence trend for a specific city, with dots representing observed values and lines indicating fitted curves based on linear or exponential functions, depending on the best-fit distribution. Notably, Huzhou and Shaoxing each have an exceptionally high data point, possibly indicating instances of human-to-human transmission. **(C)** Annual percentage changes in SFTS incidence rates by county in Zhejiang Province from 2011 to 2023. The map displays the change of the incidence rate per year for each county (p<0.05), with colors indicating the direction and magnitude of the change. Red indicates an increase in incidence, blue indicates a decrease, and the intensity of the color represents the magnitude of the change. The slopes of the incidence rates over the years were derived using the ordinary least squares regression method.

**Figure S3. Annual trend of geographic variables.**


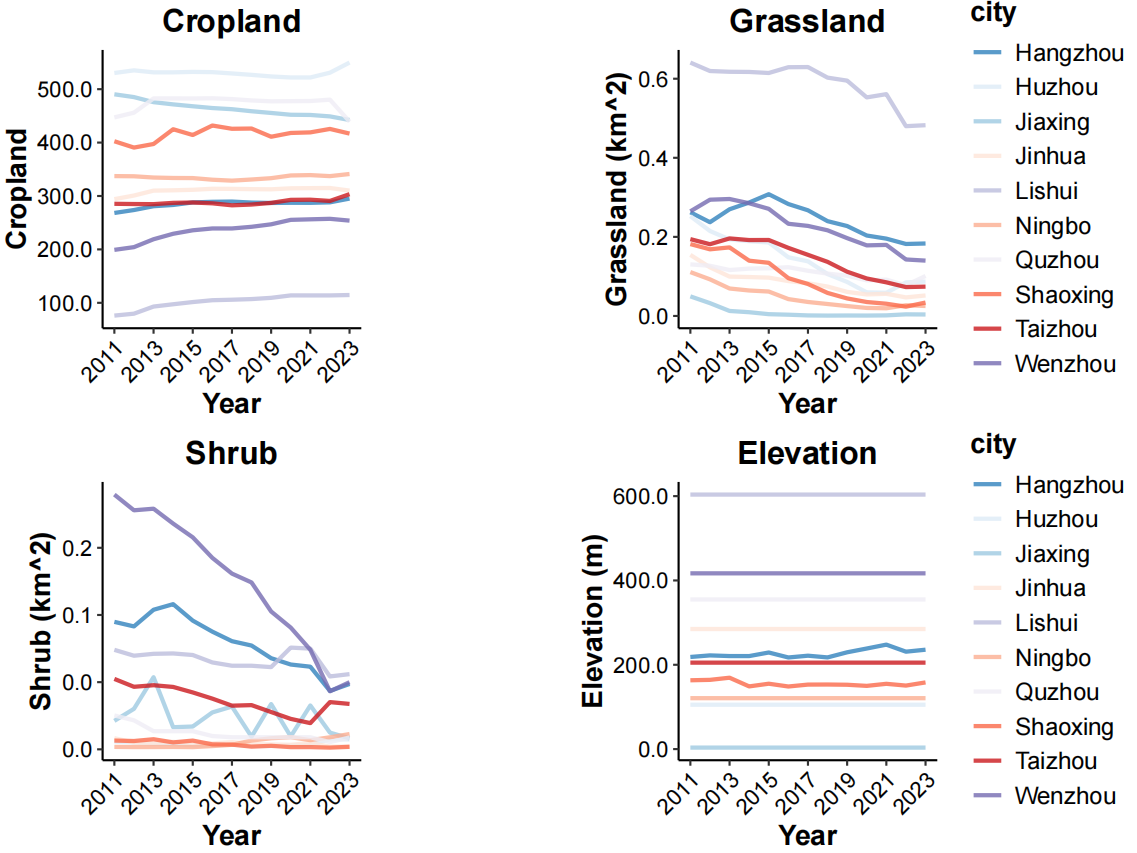


**Figure S4. The lagged and cumulative effects of climate variables within six months**

By adding each climate variable with different lag or cumulative effects to the baseline model separately, we calculated the relative risks from the univariate models. The lagged or cumulative climatic variables with the largest effect were included in the full model. The climatic covariates included in the final model were: average temperature, lagged 2-month monthly precipitation, lagged 3-month relative humidity, and 1-month cumulative sunshine duration


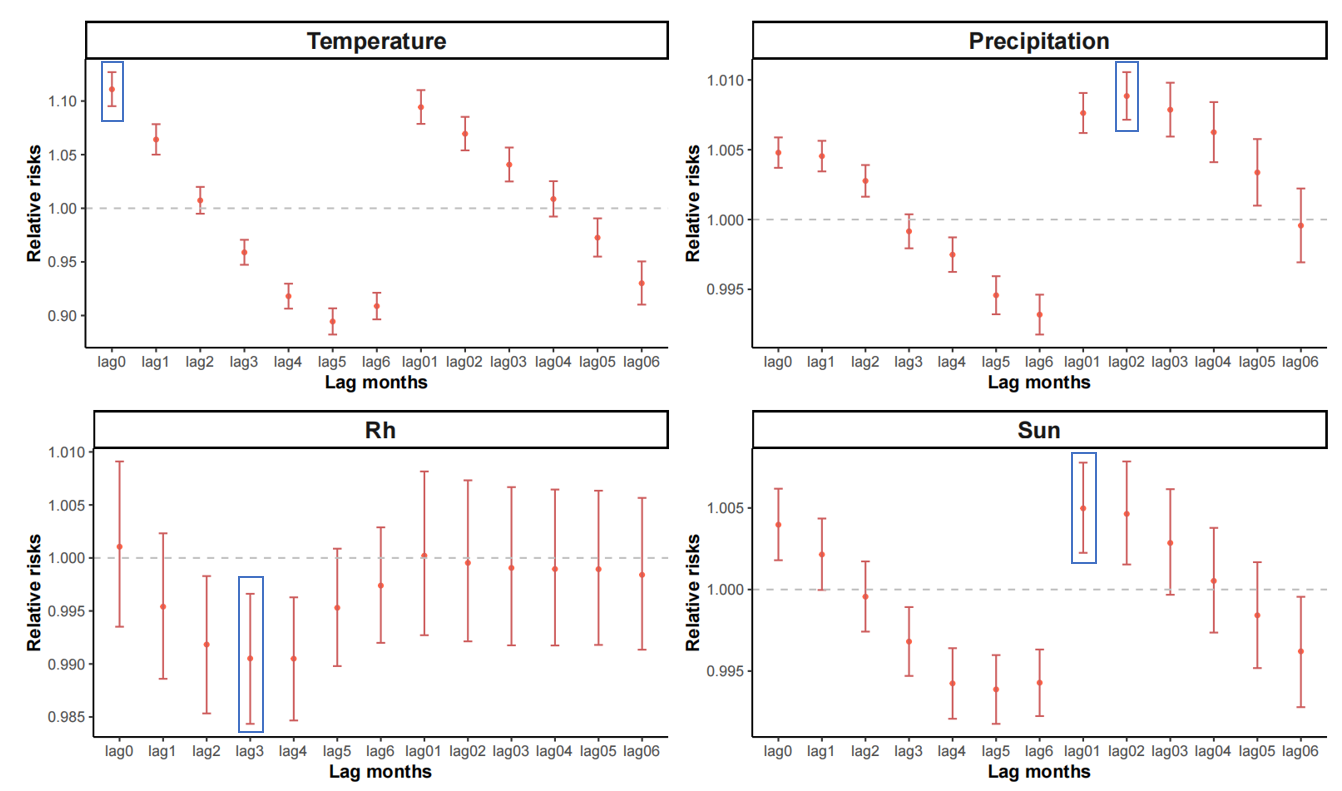


notation: Lag2 represents the climate effect from two months before; lag02 represents the cumulative effect (the moving average) of the current month, previous month, and two months before, etc. The lagged or cumulative climatic variables with the largest effect are marked in blue square.

**Figure S5. Selecting the best-fitting type for each covariate**

The covariates selected for the next step of analysis were: the nonlinear effects of elevation, nonlinear effects of forest area, nonlinear effects of grassland area, logarithm of cropland area, nonlinear effects of shrubland area, nonlinear effects of average monthly temperature, relative humidity, and sunshine duration, logarithm of monthly precipitation, logarithm of urbanization level, logarithm of transportation routes, nonlinear effects of in-migration index.

The nonlinear effects of covariates were modeled by a second-order random walk. This approach captures complex nonlinear changes by considering the relationship between adjacent points in the time series data without explicitly specifying the form of the trend. The second-order random walk model is defined as $x_{s}(t)=2x_{s}(t-1)-x_{s}(t-2)+\epsilon_{t}, \epsilon_{t}\sim N(0\mathbf{,}\sigma^{2})$.


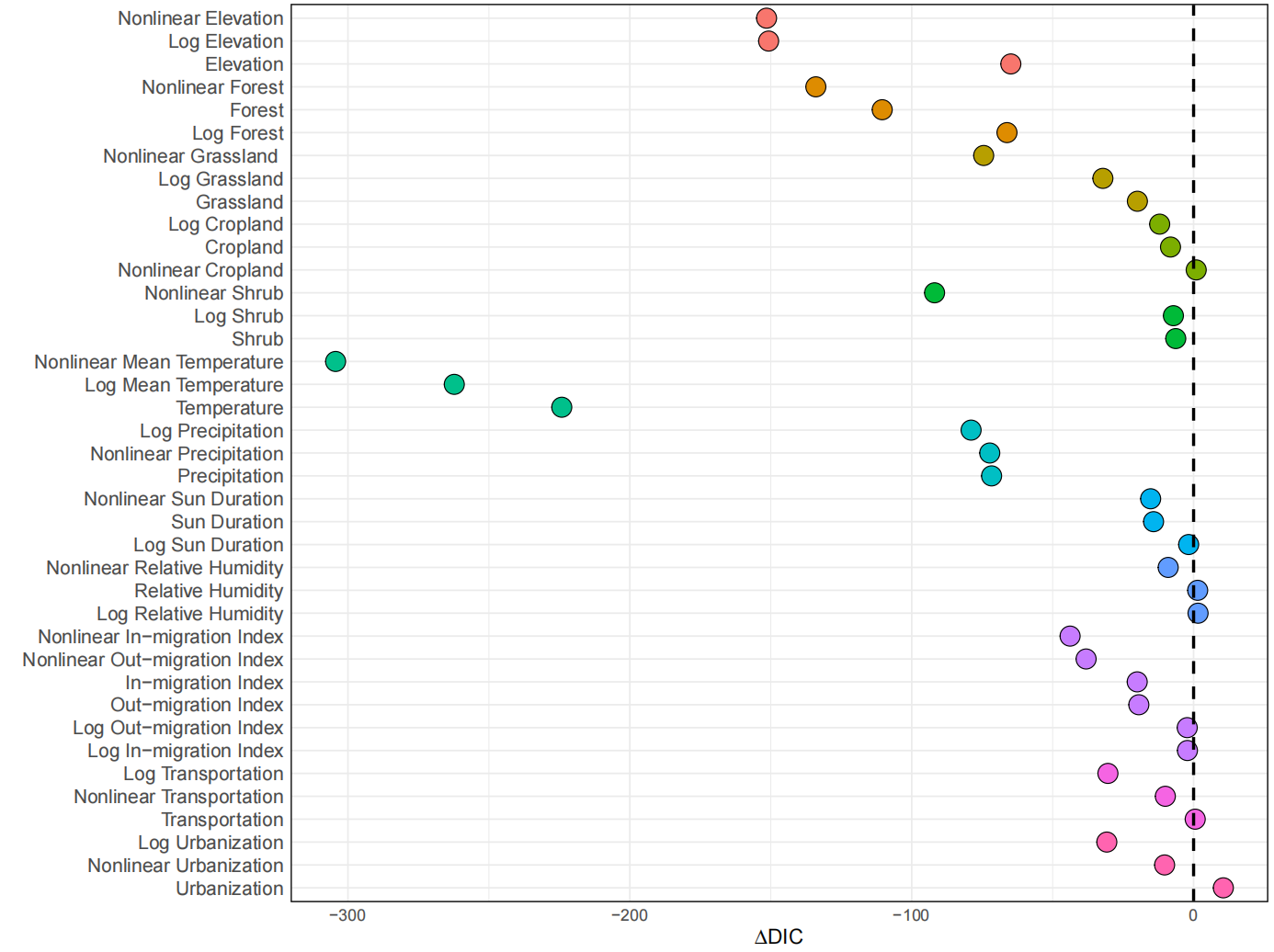


The y-axis represents different variables, and the x-axis is defined as$\Delta DIC=\mathrm{DIC}_{\mathrm{model}}-\mathrm{DIC}_{\mathrm{baseline}}$. A smaller DIC indicates better predictive performance on unobserved data. The results of different definition or functional forms for each variable are presented in the graph in ascending order from low to high.

**2.2 Addressing multicollinearity**

To address potential collinearity issues, we implemented a two-step procedure to assess and mitigate multicollinearity among covariates. First, we computed pairwise Pearson correlation coefficients between all covariates (using the optimal variable selection results from 2.1) to identify highly correlated predictor variables (Figure S6). If any pair of covariates exhibited a correlation coefficient greater than 0.7, we compared their individual associations with Severe Fever with Thrombocytopenia Syndrome (SFTS) cases. The covariate with the weaker association with SFTS, as determined by univariate regression analysis or other preliminary statistical evaluations, was removed from the model to reduce redundancy while preserving the most informative predictor.

Second, we calculated the variance inflation factor (VIF) for each remaining covariate to quantify the degree of multicollinearity within the final set of predictors. VIF values greater than 5 were considered indicative of excessive multicollinearity. Any variable exceeding this threshold was iteratively examined, and the variable with the highest VIF was removed until all remaining covariates had a VIF below 4. This step ensured that the final set of predictor variables maintained statistical independence to the greatest extent possible, thereby improving the stability and interpretability of the model estimates. (Figure S7)

Based on the above process, forest coverage was excluded from the analysis due to its collinearity with elevation.

**Figure S6. Correlation matrix for each pair of variables**

The matrix plot shows pairwise correlation coefficients between all potential covariates for the full dataset, with larger darker color denoting stronger correlation. Only one pair of covariates are strongly correlated (ρ > 0.7): the coverage of forest and elevation.


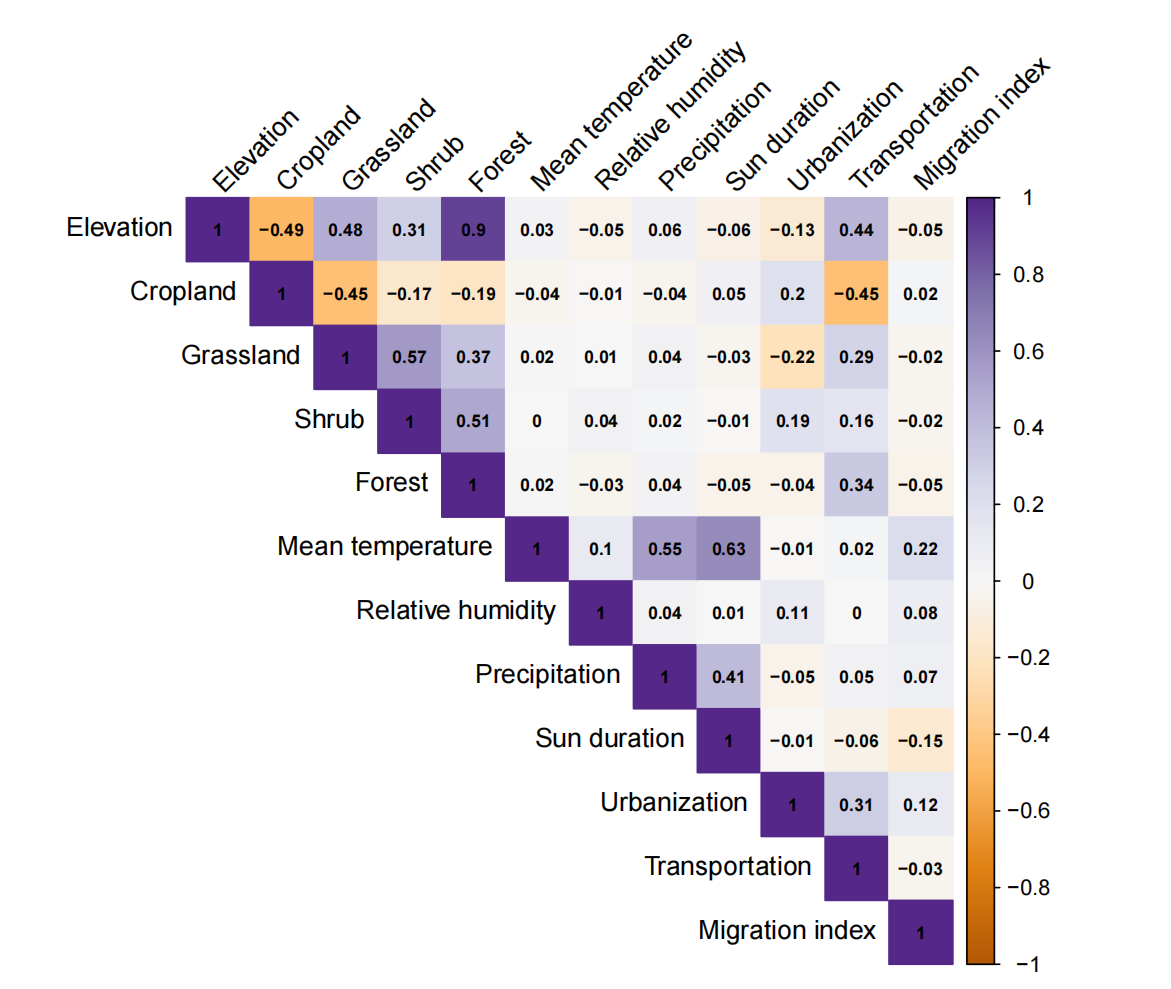


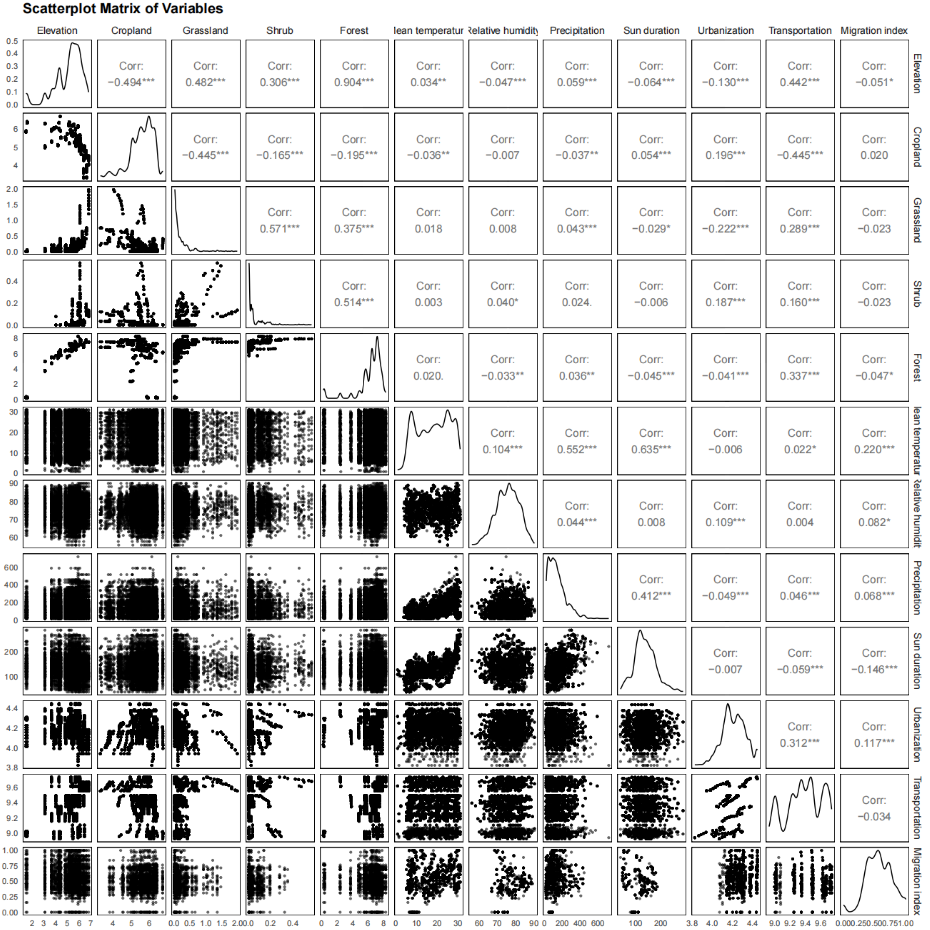


**Figure S7. Variance inflation factor (VIF) for each variable.**


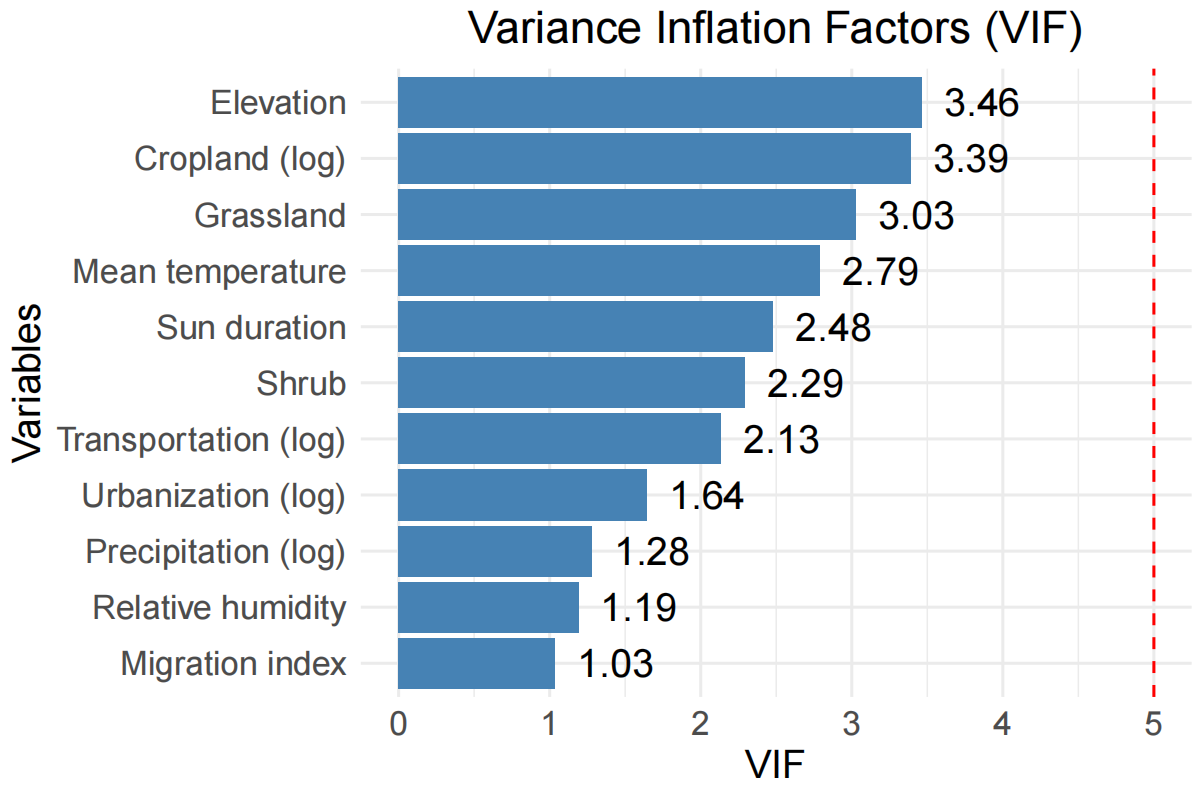


**2.3 Model structure and spatial-temporal structure assumption**

Infectious disease data often show complex temporal and spatial patterns. Bayesian spatiotemporal models can capture these patterns and account for uncertainties by incorporating spatial and temporal random effects. Within a Bayesian framework, we estimated the posterior distribution of model parameters using Integrated Nested Laplace Approximation (INLA). Monthly SFTS cases $Y_{s,t}$ were modeled as a negative binomial process:

$$Y_{s,t}\sim NegBinom\left( \mu_{s,t},n \right)$$

here 𝑠 denotes each county, 𝑡 denotes the month, $\mu_{s,t}$ represents the expected mean number of cases in county 𝑠 during month 𝑡, and n is the size (overdispersion) parameter. Utilizing Bayesian hierarchical models, we linked the observed $Y_{s,t}$​ to geographical factors, climatic factors, and social factors as follows:

$$\log\left( \mu_{s,t} \right)=\alpha+{log Pop}_{s,t}+\boldsymbol{\beta X}_{\boldsymbol{s,t}}$$

where $\alpha$ is the intercept, ${logPop}_{s,t}$ is the logarithm of population density included as an offset. $\boldsymbol{X}_{\boldsymbol{s,t}}=\left( x_{1},x_{2},x_{3},\ldots,x_{m} \right)$ and $\boldsymbol{\beta}=\left( \beta_{1},\beta_{2},\beta_{3},\ldots,\beta_{m} \right)$ are the 𝑚 explanatory variables and their coefficients.

To capture unexplained spatiotemporal variations in SFTS distribution influenced by unmeasured factors, we introduced random temporal and spatial effects. Notably, temporal and spatial effects were not mandatory components. The assumption of spatial-temporal structure and the necessity of these components was later verified through model validation.

The temporal effects included both seasonal and annual trends. Annual trends were modeled using three different functional forms, with the best-fitting model selected: (1) Linear: $\rho_{r\left( s \right),t}=c+\gamma*year index$; (2) Logarithm: $\rho_{r\left( s \right),t}=c+\gamma*log(year index)$; (3) Nonlinear: $f(year)$ is assumed to be a first-order random walk model to capture the dependencies between successive calendar months: ${\Delta\rho}_{r\left( s \right),t}=\rho_{r\left( s \right),t}-\rho_{r\left( s \right),t-1}\sim N(0\boldsymbol{,}\tau^{-1})$. The prior distribution for the precision parameter 𝜏 is specified as $\log\tau\sim\mathrm{LogGamma}(1,0.01)$. Seasonal effects were also modeled using a first-order random walk, denoted as $f(month)$.

The spatial effects were modeled using an intrinsic conditional autoregressive (ICAR) model^18^. $u_{s,y\left( t \right)}+v_{s,y\left( t \right)}$ are the spatial random effects for each year. $u_{s,y\left( t \right)}$ reflects the interaction between spatial regions. The spatial interaction between neighboring regions 𝑠 and 𝑗 can be modeled as a conditionally normal random variable, with the conditional distribution of $u_{s}$ given as:

$$u_{s}\mid{\{u}_{j,j\neq s}\}\sim N\left( \frac{1}{n_{s}}\sum_{s\sim j} u_{j}, \frac{\sigma_{s}^{2}}{n_{s}} \right)$$

where 𝑠∼𝑗 indicates neighboring regions, $n_{s}$ is the number of neighbors adjacent to 𝑠. The conditional expectation of $u_{s}$​ is the weighted average of its neighbors, $\frac{1}{n_{s}}\sum_{s\sim j} u_{j}$, with the conditional variance proportional to the number of neighbors $n_{s}$​^19^. By specifying the local conditional distribution of each random variable, a global or joint distribution can be constructed to form a Gaussian Markov random field (GMRF), where each variable's conditional distribution depends only on its neighbors. The joint distribution is constrained by $\sum u_{s}=0$ ^19^. $v_{s,y\left( t \right)}$​ reflects non-spatial heterogeneity, explaining the individual-level variation in regions and can be modeled by a normal distribution $v_{s}\sim N(0\boldsymbol{,}\theta)$. The prior distributions for the precision parameters $\sigma\mathrm{and} \theta$ are specified as LogGamma(1, 0.01).

Following a meta-modeling framework, we systematically evaluated models with varying spatiotemporal structures. The table below summarizes the tested models, incorporating different combinations of seasonal, annual, and spatial effects. Model validation was then conducted to assess their performance.

**Table S2. Summary of Tested Models with Varying Spatiotemporal Structures**

| **Model No.** | **Seasonal effect** | **Annual effect** | **Spatial effect** |
| --- | --- | --- | --- |
| 1 | f(month, model='rw1') | NA | NA |
| 2, 3, 4 | NA | f(year, model='rw1')/year/ log year | NA |
| 5 | NA | NA | f(region) |
| 6, 7, 8 | NA | f(year, model='rw1')/year/ log year | f(region) |
| 9 | f(month, model='rw1') | NA | f(region) |
| 10, 11, 12 | f(month, model='rw1') | f(year, model='rw1')/year/ log year | NA |
| 13, 14, 15 | NA | f(year, model='rw1')/year/ log year | f(region) |
| 16, 17, 18 | f(month, model='rw1') | f(year, model='rw1')/year/ log year | f(region) |

**2.4 Validation**

To prevent overfitting and ensure the reliability of our results, we conducted a comprehensive validation of candidate models with different random effect structures using multiple approaches. First, we visually assessed model fit by plotting predicted and observed values. Second, we performed five-fold cross-validation to evaluate predictive error. Third, we examined the extrapolative ability of the models by training them on datasets of varying time lengths and assessing their performance on a holdout test set. Additionally, we calculated multiple model information criteria, including the Watanabe-Akaike Information Criterion (WAIC) and Deviance Information Criterion (DIC). The final model was selected based on an integrated evaluation of all these results. Given that we tested 18 models, we first summarize the key findings and then present the most representative results (Figure S8-10).

Validation results suggest that the inclusion of seasonal effects (f(month, model='rw1')) and nonlinear temporal trends (f(year, model='rw1’)) led to overfitting. While nonlinear time effects improved in-sample fit, their complexity reduced generalizability. As a result, we removed the seasonal component and selected the log-transformed year model over the linear alternative, as it demonstrated better performance based on WAIC and DIC values. Introducing the spatial effect f(region) provided minor improvements in predictive accuracy, but its impact was relatively modest compared to models without spatial effects. To balance model complexity and predictive performance, we opted to exclude f(region) in the final model. Our final model structure incorporated only the trend term *log year*, excluding both seasonal and spatial effects.

**Figure S8. Validation results for model with varying spatiotemporal structures: case 1**

The validation results for the model with spatiotemporal structures *f(month, model='rw1')+ f(year, model='rw1')+ f(region)* are presented below. Panel A shows an almost perfect fit to the data, capturing subtle trend changes. In Panel B, despite the model's strong performance on the training set, its predictive power is poor. And due to the complexity of the model, the variance is excessively large, leading to very wide confidence intervals. Panel C shows the cross-validation results, where the model exhibits the largest prediction error among three cases (Figure S6-8). Panel D shows similar results to Panel A, indicates a good model fit. Overall, the model appears to suffer from overfitting.

The inclusion of seasonal effects *f(month,model=′rw1′)* and nonlinear time trends *f(year,model=′rw1′)* in the model yields results similar to the results in Case 1, where the improved fit for the training data came at the cost of generalizability. As a result, we opted to remove the seasonal component and chose the simpler annual component (*log year* or *year*). Since the results of the log-transformed year model and the linear model were indistinguishable, we chose the log-transformed year model due to its better performance, as indicated by WAIC and DIC values.


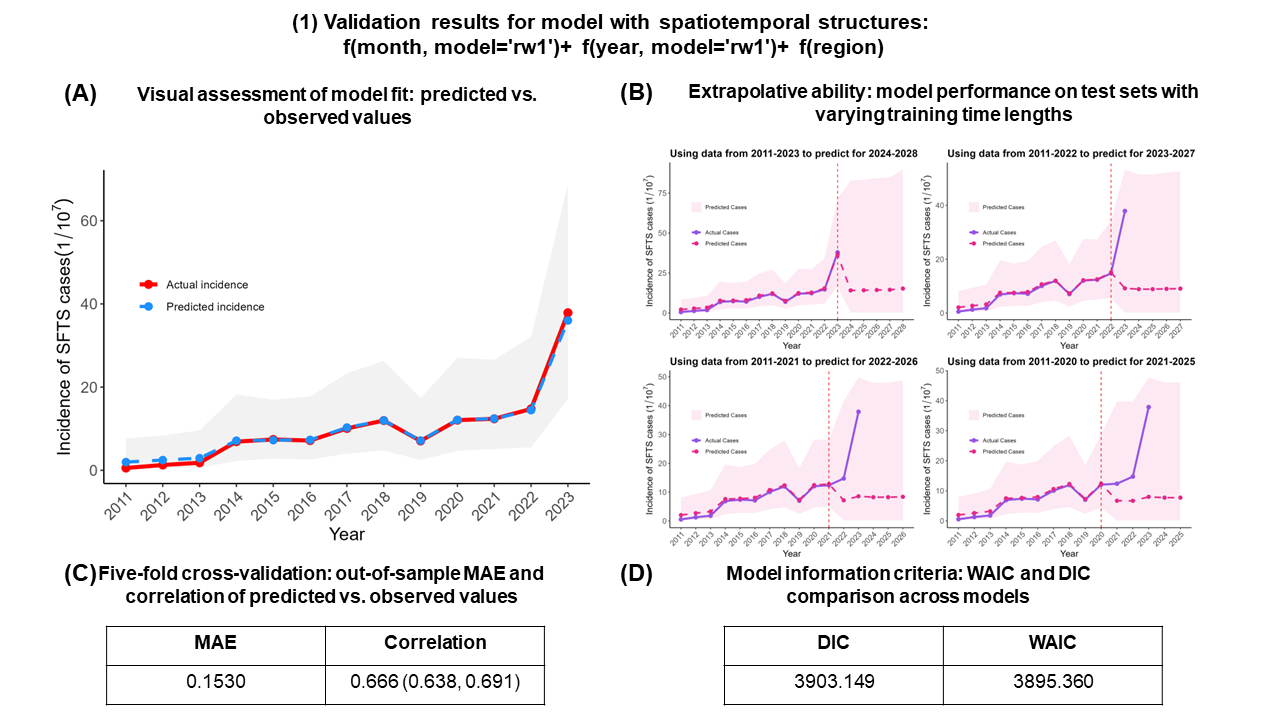


**Figure S9. Validation results for model with varying spatiotemporal structures: case 2**

The validation results for the model with spatiotemporal structures *log year+ f(region)* are presented below. Panel A demonstrates a reasonable and adequate fit to the data. In Panel B, both the training and test sets show good and consistent performance, with confidence intervals sufficiently covering the plausible predicted values without being overly wide, thus providing valuable information. Panel C illustrates the cross-validation results, where the prediction error is significantly smaller than that in Case 1. Panel D displays results similar to those in Panel A, indicating a solid model fit. Overall, the model appears robust and reasonable.

In general, this model represents a solid choice. However, the necessity of including *f(region)* remains uncertain, so we conducted an analysis for Case 3.


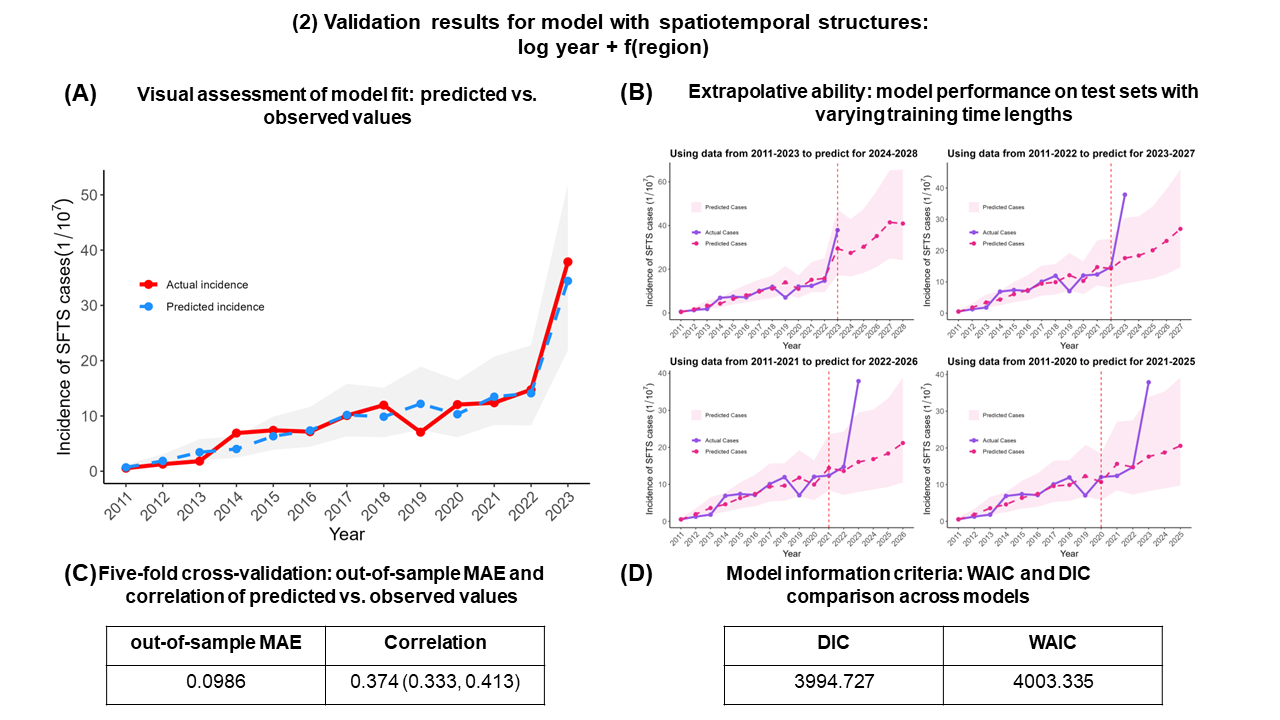


**Figure S10. Validation results for model with varying spatiotemporal structures: case 3**

The results of Case 3 were nearly the same as Case 2, except that the confidence intervals were narrower. Introducing the spatial effect *f(region)* yielded minor improvements in predictive accuracy, but its influence was relatively modest compared to models without spatial effects. To balance model complexity and predictive performance, we chose to exclude *f(region)* in the final model. Consequently, our final model structure incorporates only the trend term, the log-transformed year, while excluding both seasonal and spatial effects.


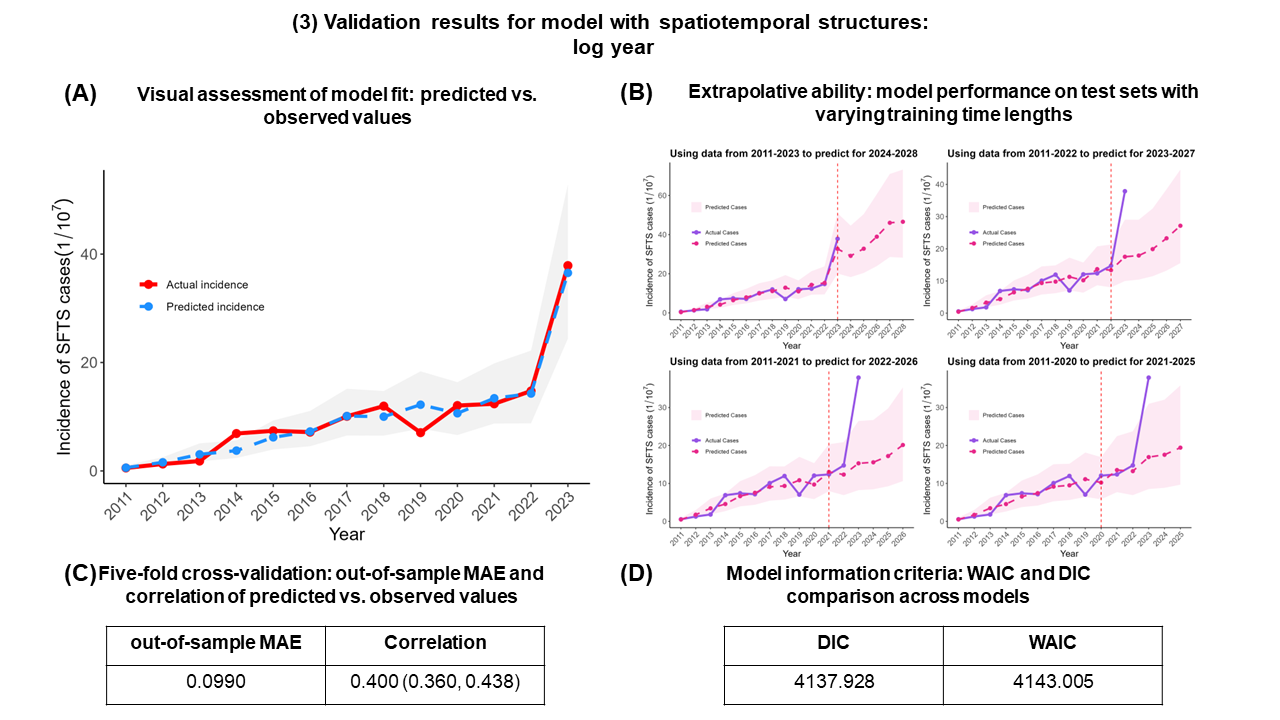


**Figure S11. Effects of socio-environmental, geographical, and climatic drivers on SFTS incidence stratified by regions**

To determine if the driving factors vary among the southern, central, and northern regions, we performed a region-specific stratified analysis.


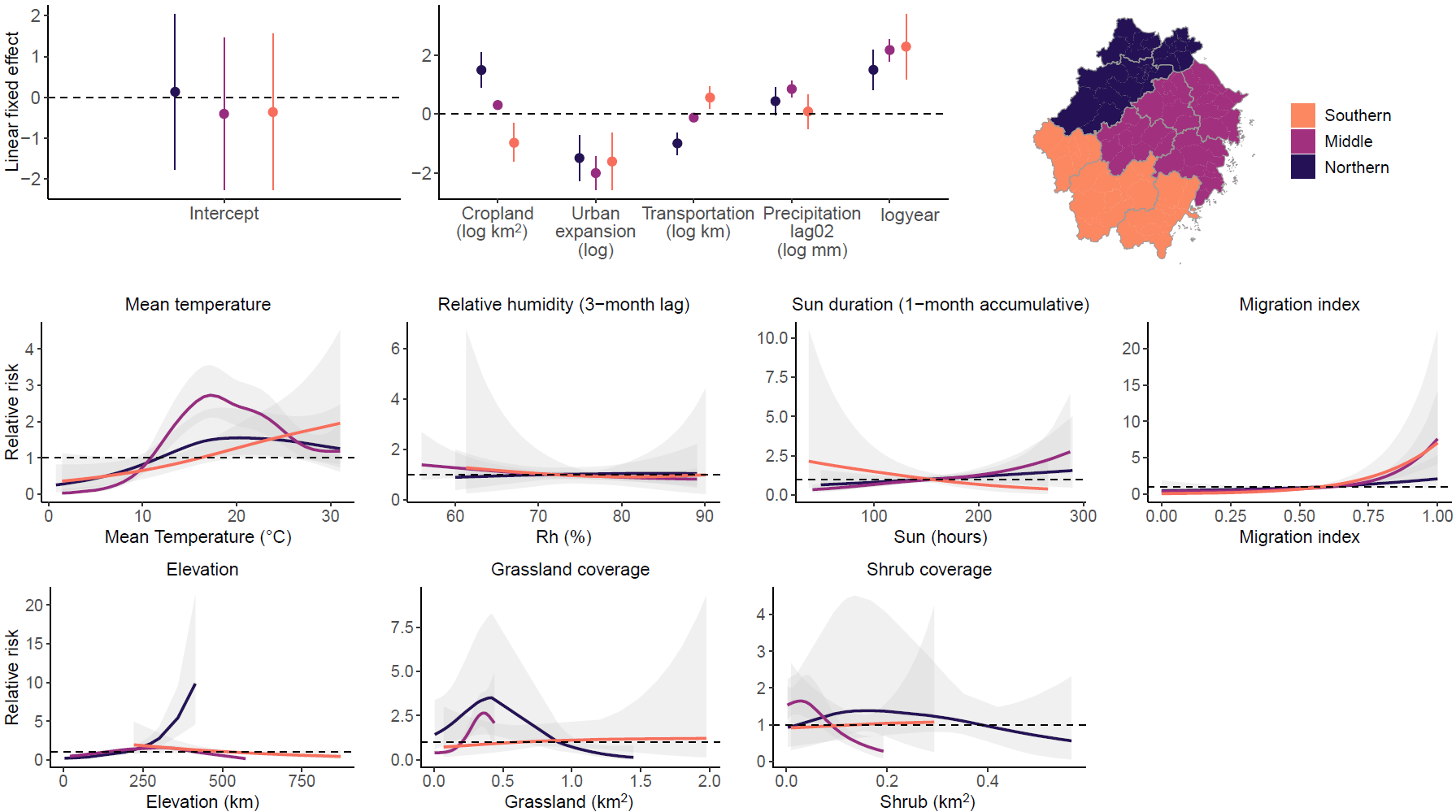


The subplots display the posterior marginal linear fixed effects and nonlinear effects of various covariates on SFTS incidence (details provided in the Methods section). The linear fixed effects are presented as risk ratios, indicating the proportional change in risk for a one-unit change in the covariate. Points and error bars in the figure represent posterior marginal means and their 95% credible intervals. The nonlinear marginal effects are specified using a second-order random walk (see Methods for details) and are shown as relative risk, where lines and bands indicate posterior means and their 95% credible intervals.

**Figure S12. Proportion of top 10 counties with highest SFTS cases**

The proportion of top 10 counties with the highest case counts across different cities shows a notable increase in Hangzhou, suggesting a rising risk of SFTS in urban centers. Historically, SFTS cases were predominantly reported in rural areas. However, the disease has now established a transmission cycle within cities, highlighting the need for increased attention to urban disease risks in the future.

Additionally, the total number of cases in the top 10 most affected regions, as a proportion of all reported cases, has gradually increased from 1% to approximately 15%. This trend indicates that SFTS cases were previously more dispersed, with no significant localized outbreaks (i.e., even the most affected regions had only a few cases, resulting in a low proportion). In contrast, the current distribution exhibits greater heterogeneity, with cases now concentrated in specific high-incidence areas.


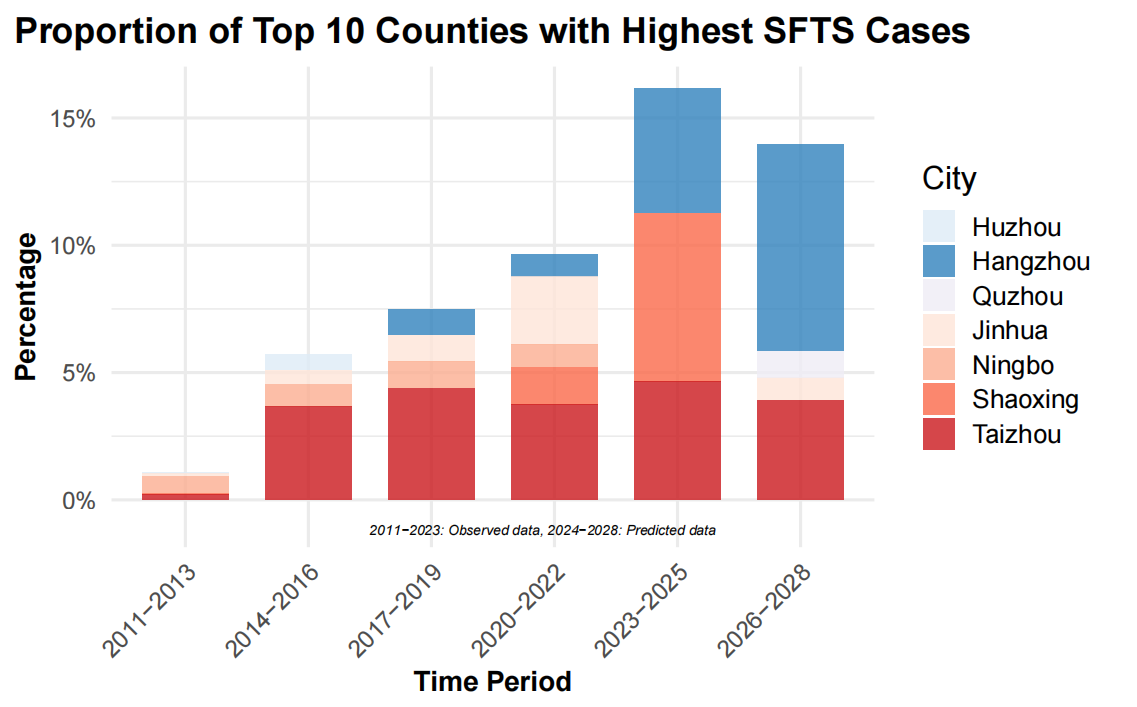


**Supplementary Text 3. Statistical analyses**

**3.1 Details in measuring the predictive influence of covariates via block cross-validation**

Using structured predictive tests to assess the effectiveness of hypothesis-driven models in predicting new data enhances the generalizability of inferred relationships. Therefore, combining explanatory and predictive approaches can strengthen inference. To estimate the influence of individual covariates on the spatiotemporal variability of SFTS incidence rates, we conducted block cross-validation experiments. These experiments calculated the out-of-sample prediction error (OOS) for the baseline model, the full model, and models where each covariate was excluded in turn. Block cross-validation, which randomizes in spatial or temporal structured blocks, is more suitable for spatially dependent phenomena such as infectious disease incidence than fully randomized approaches. In each experiment, the dataset was divided into 5 folds at county-year combination blocks using a spatiotemporal block-holdout design, and an 80%-20% train-test split (i.e., 5-fold cross-validation) was used to generate out-of-sample predictions for each model. Prediction errors, representing the differences between observed and predicted values, were summarized as mean absolute error (MAE). Each experiment was repeated 10 times. The mean and 95% confidence interval were calculated from these 10 repetitions. This design tested the contribution of each covariate to model prediction.

**3.2 The calculation for the variance explained by each type of variable**

The explained variance in the models is measured by calculating the proportion of the total variance (or deviance) in the outcome variable that is accounted for by each predictor variable. This can be done through adjusted R-squared values, partial R-squared values, or deviance explained. The usual definition of R² (variance of the predicted values divided by the variance of the data) has a problem for Bayesian fits, as the numerator can be larger than the denominator. Gelman et al^20^ proposed an alternative definition for Bayesian R², which was similar to one that has appeared in the survival analysis literature: calculated as the predicted values divided by the variance of predicted values plus the expected variance of the errors:

$\mathrm{Bayesian}R^{2}=\frac{V_{n=1}^{N}y_{n}^{\text{pred}}}{V_{n=1}^{N}y_{n}^{\text{pred}}+\mathrm{var}_{\text{res }}}$,

where the expected residual variance $\mathrm{var}_{\text{res }}=\left( \sigma^{2} \right)$, predicted values $y_{n}^{\mathrm{pred}}=$ $E\left( y\mid X_{n},\theta\right)$.

We quantified the proportion of variance explained by each covariate type based on the definition of Bayesian R². The calculation for the variance proportion attributed to factor A is given by $\frac{R_{full model}^{2}-R_{model excluded factor type A}^{2}}{R_{full model}^{2}}$. For example, the variance explained by geographical variables was $\frac{R_{full model}^{2}-R_{model excluded all geographical variables}^{2}}{R_{full model}^{2}}$. In this way, we can have an intuitive understanding of the relative significance of different types of influencing drivers. The results were shown in Figure S13.

**Figure S13. Proportion of variance explained by each type of variables**

We assessed the variance explained by each type of variables, giving a clearer perspective on their relative significance. Detailed methodology can be found in Supplementary Text 3.2.


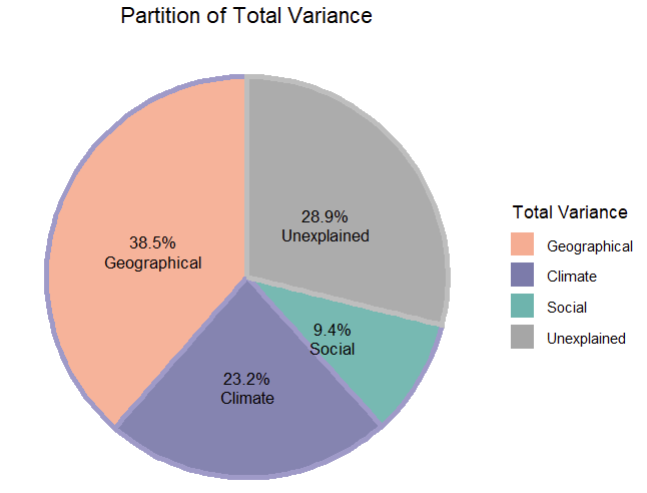


**Supplementary Text 4.1 Sensitivity Analysis Using Zero-Inflated Negative Binomial Model**

To assess the robustness of model specification and evaluate potential zero inflation in the outcome distribution, we additionally fitted a zero-inflated negative binomial (ZINB) regression model. Although zero case counts were observed in certain county-month observations, exploratory evaluation suggested that these zeros primarily reflected low-incidence transmission settings rather than a distinct structural zero-generating process.

The ZINB model was specified with the same fixed effects and random effects structure as the primary negative binomial (NB) model. Model fit statistics and parameter estimates were compared between the two specifications.

Results indicated that effect sizes, directions of association, and statistical significance were highly consistent across the NB and ZINB models. Model comparison metrics showed minimal differences, suggesting that inclusion of a zero-inflation component did not materially improve model performance. These findings support the robustness of the primary NB model specification.

**Table S3. Comparison of Model Fit Statistics Between Negative Binomial (NB) and Zero-Inflated Negative Binomial (ZINB) Models**

**Table Note:**
Model comparison includes Deviance Information Criterion (DIC), Watanabe-Akaike Information Criterion (WAIC), and logarithm of the pseudo marginal likelihood. Lower DIC and WAIC values indicate better model fit. The NB and ZINB models were fitted using the same covariates and random effects structure. Differences in model fit were minimal, indicating that inclusion of a zero-inflation component did not substantially improve model performance.

|  | dic | waic | logscore | lpml |
| --- | --- | --- | --- | --- |
| ZINB | 4142.487 | 4147.618 | 0.233232 | -0.23323 |
| NB | 4137.933 | 4142.961 | 0.232969 | -0.23297 |

**Figure S14. Effect Estimates from the Zero-Inflated Negative Binomial (ZINB) Model**


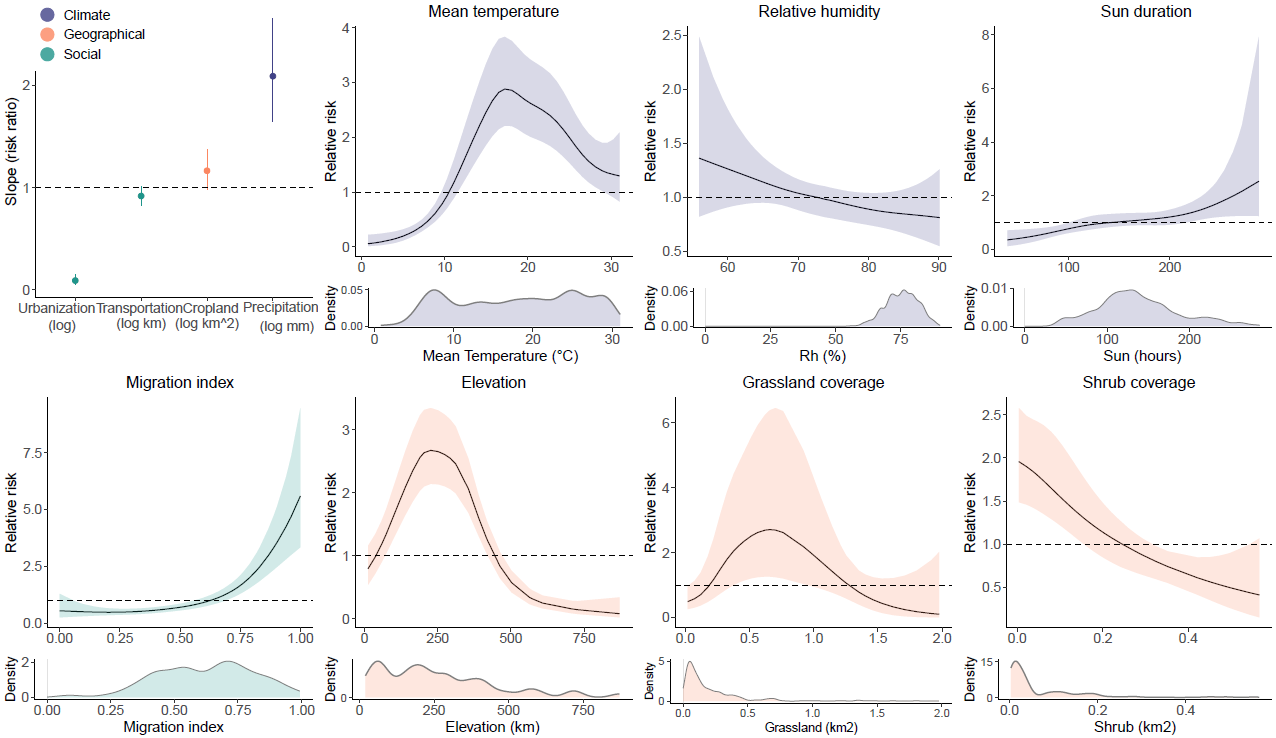


**Figure Note:**
Forest plot showing estimated incidence risk ratios (RRs) and 95% credible intervals for environmental, climatic, and social covariates under the ZINB specification. The direction and magnitude of associations were consistent with those obtained from the primary negative binomial model, indicating robustness of the findings to alternative model specifications.

**4.2 Sensitivity analysis for projecting SFTS burden under future socio-environmental and climatic changes**

We observed that CMIP6 temperature projections indicated a decrease in temperatures for 2024 compared to 2023. However, actual meteorological data up to August 30, 2024, show that temperatures in 2024 are higher than in 2023, suggesting potential inaccuracies in the CMIP6 projections. This may have led to an underestimation of the projected SFTS disease burden. To address this uncertainty, we conducted a sensitivity analysis by proportionally adjusting future temperature predictions based on the linear upward trend in historical annual temperatures.

**Figure S15. Sensitivity analysis for projecting SFTS burden under future socio-environmental and climatic changes**


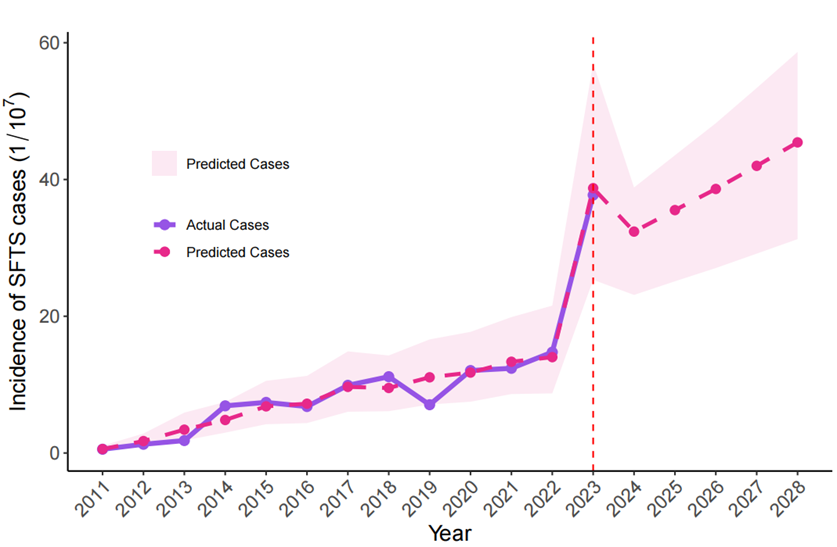


**4.3 Sensitivity analysis of the transmission window and spatial spread pattern of SFTS in Zhejiang, China using forecasting models trained without 2023 data**


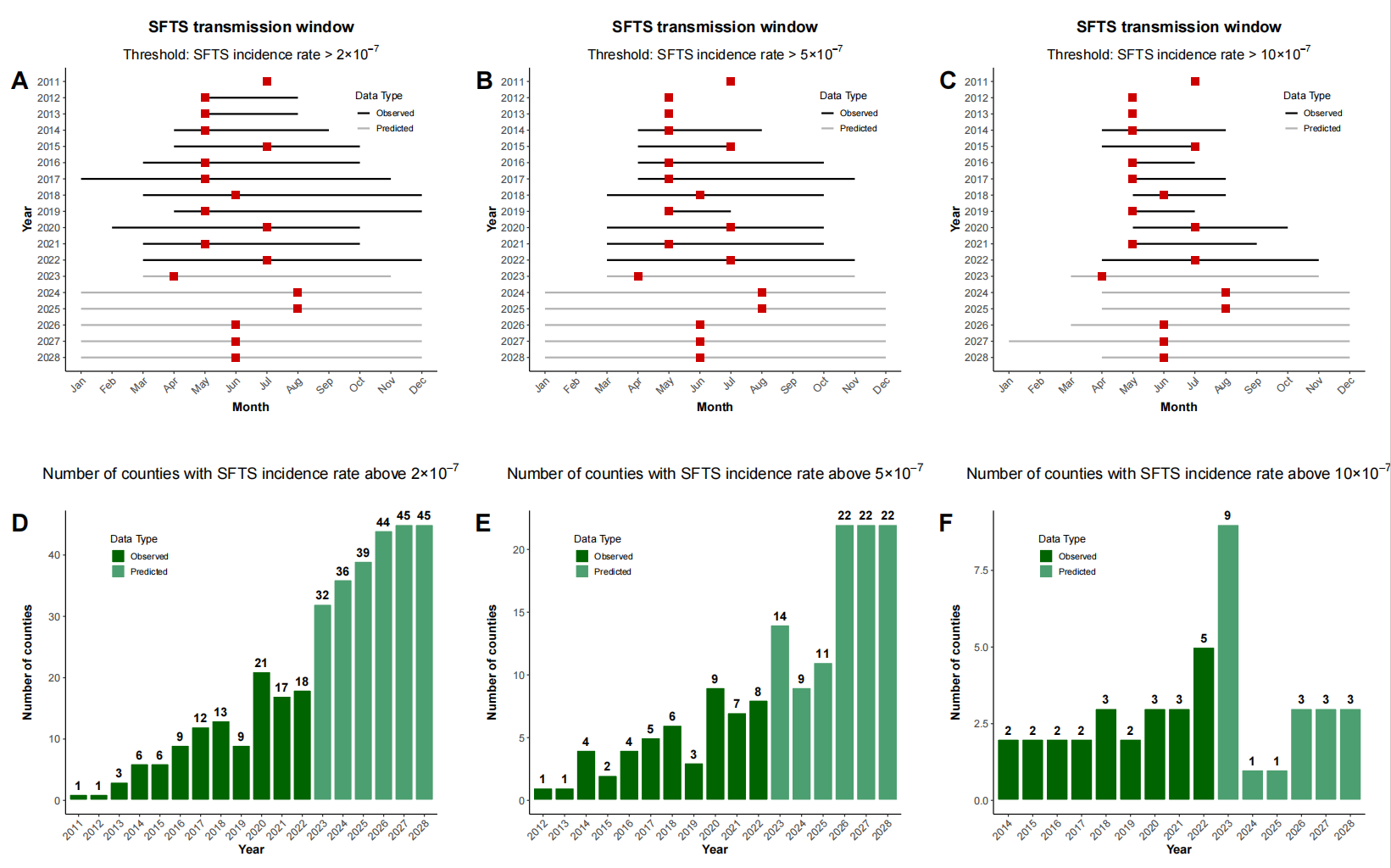


**Figure S16. Sensitivity analysis of the transmission window and spatial spread pattern of SFTS in Zhejiang, China using forecasting models trained without 2023 data**

**Supplementary Text 4.4 Sensitivity Analysis for Temporal Interpolation**

To assess the robustness of the within-year stability assumption for annual variables, we performed a sensitivity analysis using natural cubic spline interpolation to generate smooth monthly estimates from annual values. The spline-based specification allowed gradual intra-annual variation in socio-environmental indicators.

Effect estimates, direction of associations, and model fit statistics were highly consistent with those obtained under the primary monthly-expanded specification. These findings indicate that the assumption of within-year stability did not materially affect model inference.

Table S4. Sensitivity Analysis Comparing Monthly Expansion and Spline-Interpolated Annual Covariates

|  | dic | waic | logscore | lpml |
| --- | --- | --- | --- | --- |
| Monthly Expansion | 4137.933 | 4142.961 | 0.232969 | -0.23297 |
| Spline-Interpolated | 4138.517 | 4143.284 | 0.233114 | -0.233115 |

**Supplementary Text 4.5 Sensitivity Analysis Using Alternative Season Definitions**

To evaluate the robustness of epidemic season estimation, we conducted additional sensitivity analyses using a distribution-based definition of epidemic season that does not rely on predefined incidence thresholds.

For each calendar year, monthly SFTS case counts were first ordered chronologically. We then calculated the cumulative proportion of annual cases over time. The epidemic season was defined as the shortest continuous interval within the year during which the cumulative case numbers accounted for more than 70%, 80%, or 90% of the total annual cases. The start and end points of this interval were determined as the earliest and latest months satisfying the specified cumulative proportion criterion.

Seasonal indicators derived from this definition—including season onset time, season end time, and total season duration—were compared with those obtained from the incidence-based thresholds (2, 5, and 10 cases per 100 million population-month). Consistency was assessed by examining whether projected temporal trends and climate-driven seasonal extensions remained directionally stable across definitions.


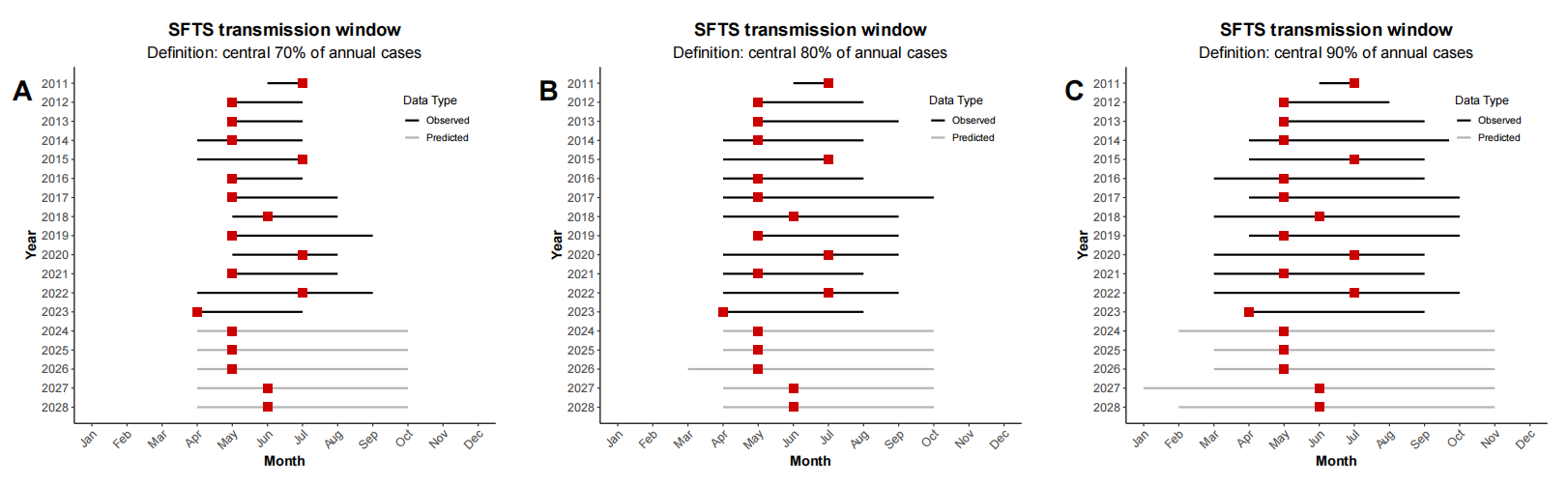


**Figure S17. Sensitivity analysis of SFTS epidemic season definitions using cumulative case distribution–based criteria**

**Caption:**

Comparison of epidemic season timing and duration derived from cumulative case distribution–based definitions (>70%, >80%, and >90% of annual cases) and incidence threshold–based definitions (2, 5, and 10 cases per 100 million population-month). Panels (A–C) show season onset, season end, and season duration under different definitions, respectively. Although absolute season duration varies slightly depending on the definition applied, the temporal trends and projected seasonal extensions under climate change scenarios remain consistent across all criteria.

**References**

1. Ministry of Health of People's Repubic of China. Guideline for prevention and treatment of sever fever with thrombocytopenia syndrome (2010 vesrion). Chinese Journal of Clinical Infectious Diseases. 2011;04(04)

2. Wang Y, Cai H, Yan Y, et al. Regime shifts in the thermal dynamics of offshore China due to accelerated global warming. Sci Total Environ 2024; 949: 174882.

3. Gibb R, Colón-González FJ, Lan PT, et al. Interactions between climate change, urban infrastructure and mobility are driving dengue emergence in Vietnam. Nat Commun 2023; 14(1): 8179.

4. Shouzhang P. 1 km multi-scenario and multi-model monthly temperature data for China (2021-2100). In: National Tibetan Plateau Data C, editor.: National Tibetan Plateau Data Center; 2024.

5. Watanabe S, Kanae S, Seto S, Yeh PJ-F, Hirabayashi Y, Oki T. Intercomparison of bias-correction methods for monthly temperature and precipitation simulated by multiple climate models. Journal of Geophysical Research: Atmospheres 2012; 117(D23).

6. Yang J, Huang X. The 30 m annual land cover dataset and its dynamics in China from 1990 to 2019. In Earth System Science Data 2021; 13(8): 3907-25.

7. European Space Agency S. Copernicus Global Digital Elevation Model. Distributed by OpenTopography. 2021. https://doi.org/10.5069/G9028PQB.

8. Wenzhou Traffic and Transportation Bureau. Wenzhou's Transportation: Bold Progress and Rapid Development Over the Past Two Decades. https://wzjt.wenzhou.gov.cn/art/2023/8/2/art_1243903_58927906.html

9. Hassell JM, Begon M, Ward MJ, Fèvre EM. Urbanization and Disease Emergence: Dynamics at the Wildlife-Livestock-Human Interface. Trends Ecol Evol 2017; 32(1): 55-67.

10. Deng B, Rui J, Liang SY, et al. Meteorological factors and tick density affect the dynamics of SFTS in jiangsu province, China. PLoS Negl Trop Dis 2022; 16(5): e0010432.

11. Sun J-M, Wu H-X, Lu L, et al. Factors associated with spatial distribution of severe fever with thrombocytopenia syndrome. Science of The Total Environment 2021; 750: 141522.

12. Wu H, Wu C, Lu Q, Ding Z, Xue M, Lin J. Spatial-temporal characteristics of severe fever with thrombocytopenia syndrome and the relationship with meteorological factors from 2011 to 2018 in Zhejiang Province, China. PLoS Negl Trop Dis 2020; 14(4): e0008186.

13. Miao D, Liu MJ, Wang YX, et al. Epidemiology and Ecology of Severe Fever With Thrombocytopenia Syndrome in China, 2010‒2018. Clin Infect Dis 2021; 73(11): e3851-e8.

14. Liu K, Zhou H, Sun RX, et al. A national assessment of the epidemiology of severe fever with thrombocytopenia syndrome, China. Sci Rep 2015; 5: 9679.

15. Jiang X, Wang Y, Zhang X, et al. Factors Associated With Severe Fever With Thrombocytopenia Syndrome in Endemic Areas of China. Front Public Health 2022; 10: 844220.

16. Stoddard ST, Morrison AC, Vazquez-Prokopec GM, et al. The role of human movement in the transmission of vector-borne pathogens. PLoS Negl Trop Dis 2009; 3(7): e481.

17. Findlater A, Bogoch, II. Human Mobility and the Global Spread of Infectious Diseases: A Focus on Air Travel. Trends Parasitol 2018; 34(9): 772-83.

18. Besag J, York J, Mollié A. Bayesian image restoration, with two applications in spatial statistics. Annals of the Institute of Statistical Mathematics 1991; 43: 1-20.

19. Morris M, Wheeler-Martin K, Simpson D, Mooney SJ, Gelman A, DiMaggio C. Bayesian hierarchical spatial models: Implementing the Besag York Mollié model in stan. Spat Spatiotemporal Epidemiol 2019; 31: 100301.

20. Gelman A, Goodrich B, Gabry J, Vehtari A. R-squared for Bayesian Regression Models. The American Statistician 2019; 73(3): 307-9.
